# tRNA Biology in Mitochondria

**DOI:** 10.3390/ijms16034518

**Published:** 2015-02-27

**Authors:** Thalia Salinas-Giegé, Richard Giegé, Philippe Giegé

**Affiliations:** 1 Institut de Biologie Moléculaire des Plantes, CNRS and Université de Strasbourg, 12 rue du Général Zimmer, F-67084 Strasbourg Cedex, France; E-Mail: thalia.salinas@ibmp-cnrs.unistra.fr; 2 Institut de Biologie Moléculaire et Cellulaire, CNRS and Université de Strasbourg, 15 rue René Descartes, F-67084 Strasbourg Cedex, France; E-Mail: r.giege@ibmc-cnrs.unistra.fr

**Keywords:** evolution, tRNA identity, tRNA import, organelle gene expression, RNase P

## Abstract

Mitochondria are the powerhouses of eukaryotic cells. They are considered as semi-autonomous because they have retained genomes inherited from their prokaryotic ancestor and host fully functional gene expression machineries. These organelles have attracted considerable attention because they combine bacterial-like traits with novel features that evolved in the host cell. Among them, mitochondria use many specific pathways to obtain complete and functional sets of tRNAs as required for translation. In some instances, tRNA genes have been partially or entirely transferred to the nucleus and mitochondria require precise import systems to attain their pool of tRNAs. Still, tRNA genes have also often been maintained in mitochondria. Their genetic arrangement is more diverse than previously envisaged. The expression and maturation of mitochondrial tRNAs often use specific enzymes that evolved during eukaryote history. For instance many mitochondria use a eukaryote-specific RNase P enzyme devoid of RNA. The structure itself of mitochondrial encoded tRNAs is also very diverse, as e.g., in Metazoan, where tRNAs often show non canonical or truncated structures. As a result, the translational machinery in mitochondria evolved adapted strategies to accommodate the peculiarities of these tRNAs, in particular simplified identity rules for their aminoacylation. Here, we review the specific features of tRNA biology in mitochondria from model species representing the major eukaryotic groups, with an emphasis on recent research on tRNA import, maturation and aminoacylation.

## 1. Introduction

The understanding of mitochondrial biology has important societal implications, primarily because mitochondrial dysfunctions often result in serious disorders such as myopathies or other neuro-degenerative diseases in human [1]. Similarly, in other eukaryotes such as plants, mitochondrial (mt) mutations also result in serious dysfunctions, in particular cytoplasmic male sterility, a genetic trait widely used for agronomy [2]. Mitochondria, together with chloroplasts in some phyla, are the power stations of eukaryotic cells. They are the sites where essential energy production processes such as oxidative phosphorylation and the tricarboxylic acid cycle are performed. Mitochondria are frequently referred to as semi-autonomous because they contain genomes as well as comprehensive gene expression machineries. This occurrence strongly contributed to revitalizing the endosymbiotic model [3,4,5]. The availability of many complete mt-genome sequences has strongly established the eubacterial lineage of mitochondria. However, besides the α-proteobacterial origin of these organelles, it was also recently proposed that a “pre-mitochondrion” membrane-bound metabolic organelle that already encompassed many of the non-energy-related functions of modern mitochondria pre-existed before the α-proteobacterial endosymbiosis [6]. Contemporary mt-genomes still encode a relatively well-conserved core set of genes. It is composed of two major classes of genes encoding key components required for energy production such as subunits of the respiratory chain complexes and factors required for mt-translation, in particular tRNAs. While mt-genome structures greatly vary, gene content is not correlated with the disparity of genome sizes. It is rather gene density that varies among genomes. For example, it is remarkable that the 16.5 kb human mt-genome is 22 times smaller than *Arabidopsis* mt-genome but encodes as many as one quarter of the genes present in *Arabidopsis* [7]. The examination of the residual mt-genes in all phyla shows that the only direct or indirect function of mt-genomes is to express respiratory proteins.

Still, mt-genomes are far from being able to express all the components needed to assemble the respective complexes, in particular to sustain functional translation machineries. The remaining proteins and RNAs required, in particular tRNAs as well as all the proteins involved in the biogenesis of mitochondrial encoded tRNAs (mt-tRNAs) are coded in the nucleus, expressed in the cytosol and have to be imported into mitochondria. In view of this, it is evident that mt-biogenesis absolutely depends on nuclear encoded factors and that precise communication and regulatory processes are required between mitochondria and the nucleus for mt-biogenesis [8]. While protein import processes are comparatively well characterized [9], the understanding of tRNA import regulation, mechanisms and evolutive diversity remains a challenge [10,11,12].

Beyond the universal conservation of core mt-functions of prokaryote origin, it is remarkable that mt-gene expression relies on a wide and diverse array of specific processes that have arisen during eukaryote evolution [13,14]. Among them, mitochondria use many original processes for the expression and maturation of tRNAs. These processes involving a number of recently recognized factors are reviewed here together with the import pathways required to reach the full set of tRNAs in mitochondria as well as the original features that define the structure and function of mt-tRNAs.

## 2. The Pool of tRNAs in Mitochondria Consists of Encoded and Imported tRNAs

### 2.1. Distribution and Origin of tRNAs Encoded in Mitochondria

The increasing availability of complete mt-genomes from different species and their bioinformatics analysis allowed the identification of mitochondria-encoded tRNA genes in numerous organisms. These analyses show that mt-genomes encode a variable set of tRNAs derived from the α-proteobacterial ancestor (Table 1). The translation of the few proteins encoded in the mt-genome requires at least 20–22 different tRNAs, depending on the genetic code and the wobble rules. However, the presence of a complete minimalist set of tRNA genes encoded by the mt-genome is more an exception than a general rule [15]. The lack of tRNA genes goes from one tRNA (e.g., in the marsupial metazoan *Didelphis virginiana*) to the most extreme situations in protozoans such as trypanosomatides (e.g., *Trypanosoma brucei*, *Leishmania tarentolae*) and alveolates (e.g., apicomplexans such as *Plasmodia*, *Toxoplasma*) where the mt-genomes can be completely devoid of tRNA genes. Interestingly, species with many missing mt-tRNA genes can be closely related to species that have a full set of mt-tRNA genes [16]. For example in fungi, *Saccharomyces cerevisiae* encodes for a complete set of tRNA genes whereas *Spizellomyces punctatus* encodes for only eight mt-tRNA genes [17]. Similarly, the alga, *Nephroselmis olivacea* encodes a complete set of tRNA genes, whereas *Chlamydomonas reinhardtii* codes for only three mt-tRNA genes [18]. This indicates that the loss of tRNA genes is not consistent with the assigned phylogenetic positions and probably occurred during multiple independent events. A particularity of plants is that, mt-genomes of angiosperms (flowering plants) acquired tRNA genes of different origins during evolution [19]. For example, the native genes for tRNA^His^ and tRNA^Asn^ were lost in all angiosperms investigated so far and have been replaced by chloroplast-like genes [20]. Similarly, a tRNA^Cys^ gene of bacterial origin was acquired by horizontal gene transfer during angiosperms evolution [21]. However, the acquisition and integration of foreign tRNAs is exceptional and missing mt-tRNAs are most of the times compensated by the import of nucleus-encoded tRNAs.

**Table 1:** Overview on mitochondrially encoded tRNA genes and extent of tRNA import in mitochondria of representative eukaryotic taxonomic groups and species. The number of tRNA genes encoded by the mt-genome that are indicated, including duplicated genes. Genbank accessions and references that allowed numbers determination are indicated. The tRNAs missing and/or imported are designated by the specificity of their anticodons indicated by the amino acid one-letter code; tRNA isoacceptors are distinguished by their anticodon. References are given for cytosolic tRNAs for which import has been experimentally proven; n.d. mitochondrial genomic sequence not available. Adapted from references [15,22].

Species	tRNA Genes Encoded by the mt-Genome	tRNA Genes Missing/not Expressed	Import Demonstrated	References	
				tRNA Content	tRNA Import
**Metazoans**					
**Mammals**					
*Homo sapiens*	22	0	Q	NC_012920	[23]
*Didelphis virginiana*	21	K	K	NC_001610	[24]
**Molluscs**					
*Crassostrea gigas*	18	A, F, S		AF177226	
**Cnidarians**					
*Metridium senile*	2	all but 2		NC_000933	
**Fungi**					
**Ascomycotes**					
*Saccharomyces cerevisiae*	24	0	L, Q	NC_000933	[25,26]
**Chytridiomycotes**					
*Spizellomyces punctatus*	8	all but 8		NC_003052 NC_003061 NC_003060	
**Plants**					
**Bryophytes**					
*Marchantia polymorpha*	29	0	I^(IAU)^ , T^(AGU)^, V^(AAC)^	NC_001660 [27]	[28,29,30]
**Angiosperms**					
*Arabidopsis thaliana*	22	all but 15	F, W	NC_001284	[31,32]
*Nicotiana tabacum*	23	at least 6	A, G, V	NC_006581	[33,34,35,36]
*Solanum tuberosum*	20	at least 7	A, G, I, L, R, T, V	[37]	[38,39,40]
*Triticum aestivum*	16	14	A, G, H, I^(IAU)^, L, R, V	NC_007579	[41]
**Gymnosperms**					
*Larix leptoeuropaea*	n.d	at least 11	A, G, F, I^(IAU)^, L, K, P, S^(GCU)^, S^(UGA)^, T, V		[39]
**Chlorophytes**					
*Chlamydomonas reinhardtii*	3	all but 3	for 31 tRNAs	NC_001638	[42]
*Scenedesmus obliquus*	27	T		NC_002254	
*Nephroselmis olivacea*	26	0		NC_008239	
**Other eukaryotes**					
**Jakobides**					
*Reclinomonas americana*	26	T		NC_001823	
**Ciliophores**					
*Tetrahymena pyriformis*	10	all but 10	for 26 tRNAs	NC_000862	[43,44,45]
**Trypanosomatides**					
*Leishmania tarentolae*	0	all	all except Q^(CUG)^	[46,47,48]	
*Trypanosoma brucei*	0	all	all except initiator M and U	[22,49,50]	
**Apicomplexans**					
*Plasmodium falciparum*	0	all	C, F	NC_002375	[51]

### 2.2. Mitochondrial Import of tRNAs

Contrary to initial thoughts, tRNA import into mitochondria is widespread and the number of imported tRNAs varies across species and phyla (Table 1). An overview of the different aspects of tRNA import is presented here to supplement reviews on that topic (e.g., [10,11,12,22,52,53,54,55]). Import is obvious for organisms where mt-tRNA genes are missing while proper mt-translation has to be performed. However, experimental data also showed that organisms that encode a presumably complete set of mt-tRNAs can also import cytosolic tRNAs as demonstrated in *S. cerevisiae* and human mitochondria [23,25,26]. In these instances where imported tRNAs seem to be redundant, import can be important in certain conditions, as shown in *S. cerevisiae* with the necessity to import cytosolic tRNA^Lys(CUU)^ to decode a lysine codon under stress conditions [56]. In some cases, the import can be underestimated, e.g., the analysis on *C. reinhardtii* mitochondria showed that 31 cytosolic tRNAs are imported instead of the 22 expected ones [42]. Therefore, the functional reasons and numbers of imported tRNAs are difficult to predict and individual experimental analyses are required.

#### 2.2.1. Determinants for tRNA Import

In all the organisms studied so far, the imported tRNAs derive from cytosolic tRNAs that are also essential for cytosolic translation. Not all the cytosolic tRNAs are imported into mitochondria: some are exclusively in the cytosol and others are shared between cytosol and mitochondria. The question of how the cell discriminates between imported and cytosol-specific tRNAs has been studied in various organisms and different approaches have been used to identify determinants in imported tRNAs or antideterminants in cytosol-specific tRNAs. Results indicate that tRNA import signals are present on mature tRNAs only and that they are as diverse as the number of organisms studied [20,22]. The main reason that could explain why multiple import signals have been identified is that import determinants may depend on protein import factors interacting with tRNAs all along the import process [10]. There are only few examples where import determinants are necessary and sufficient for the *in vivo* mitochondrial or cytosol-specific localization of tRNAs, *i.e.*, for tRNA^Lys^ in *S. cerevisiae* [57,58], tRNA^Gln^ in *Tetrahymena thermophila* [58,59] and the cytosol-specific initiator tRNA^Met^ and tRNA^Sec^ in *T. brucei* [60,61]. In plants, import signals that are necessary and sufficient for mt-localization could not be identified and only mutations abolishing import have been described [62,63]. Import selectivity remains puzzling as tRNAs of identical sequence in two species can be imported in one species but not in the other. Contrary to yeast or human, plant populations of imported tRNAs are strictly complementary to those of mt-encoded tRNAs, suggesting that only cytosolic tRNAs required for translation are imported into plant mitochondria [18,63].

#### 2.2.2. Compared Levels of Imported tRNAs

The steady-state level of tRNAs imported into mitochondria corresponds to a small percentage of the total amount of cytosolic tRNAs. This means that most cytosolic tRNAs are used in the cytosolic translation machinery. Nevertheless, the proportion of tRNAs found in mitochondria varies for individual imported tRNAs. For instance, in *Leishmania tarentolae*, tRNAs are classified into three groups, mainly cytosolic, mainly mitochondrial and shared between the two compartments, according to their relative abundance in the cytosol or mitochondria [64,65]. In *T. brucei*, the quantification of the abundance of each tRNA in the cell and in mitochondria revealed that the extent of their mt-localization fluctuates between 1% and 7.5% [66]. In *S. cerevisiae*, the imported cytosolic tRNA^Lys(CUU)^ represents only 3%–5% of the total cellular amount [66,67]. In land plants, no extensive study of mt-tRNA localization has been performed. However a study on tRNA^Gly^ isoacceptors in tobacco showed that the imported tRNA^Gly(UCC)^ represents 2.5% of total tRNA^Gly(UCC)^ whereas the imported tRNA^Gly(CCC)^ represents 6.5% of total tRNA^Gly(CCC)^ [36]. In the green alga *Chlamydomonas*, an in-depth study showed that out of the 49 cytosolic tRNA isoacceptors, 31 were present within mitochondria. For 28 tRNAs, the extent of mt-localization ranged from 0.2%–26% and in contrast with other investigated organisms, three tRNAs had a mt-localization higher than 80%. In particular, tRNA^Lys(UUU)^ has an import level of 98% and is thus regarded as the first example of a nuclear-encoded tRNA exclusively found in mitochondria [42]. Remarkably, the observed steady-state levels of imported tRNAs correlate with the occurrence frequencies of the cognate codons for both mitochondrial and nuclear genes. This fine-tuning between tRNA import and the codon usage in *Chlamydomonas* seems to be the result of a co-evolutive process rather than a dynamic adaptation of cytosolic tRNA import into mitochondria [68].

#### 2.2.3. Mechanistic Insights into tRNA Import

**Figure 1:**
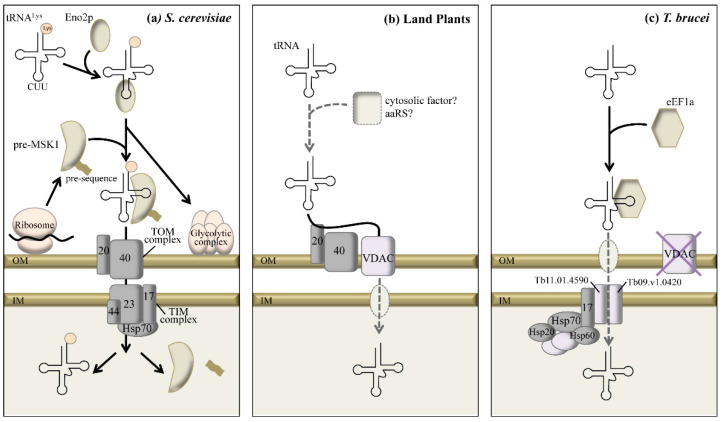
Comparative models showing the factors involved in tRNA import in mitochondria from (**a**) *S. cerevisiae*, (**b**) land plants and (**c**) *T. brucei*. Black arrows represent characterized steps of import, whereas grey dotted arrows represent tentative steps. OM and IM stand for mt-outer and inner membrane, respectively. TIM and TOM stand for translocase of the inner and outer mt-membrane. The subunits of TOM and TIM complexes are distinguished by their size given in kDa. Hsp describes different heat shock proteins, Eno2p stands for the glycolytic enzyme Enolase, aaRS for aminoacyl-tRNA synthetase, VDAC for the Voltage Dependent Anion Channel, pre-MSK1 for the precursor of the mitochondrial lysyl-tRNA synthetase, eEF1a for the eukaryotic translation elongation factor 1 alpha, and Tb for *Trypanosoma brucei*. Proteins shown in grey denote proteins belonging to the protein import machinery. Adapted from refs [10,22].

Mechanisms for tRNA import in mitochondria are complex and their complete understanding in representative organisms remains a challenge. This process can be broken up into two distinct steps. First, cytosolic tRNAs have to be deviated from the cytosolic translation machinery in order to be addressed to the mitochondrial surface. Then, tRNAs have to be translocated through mitochondrial membranes to finally reach the mt-translation machinery. During these steps, according to the channeling theory, the tRNA is not “free” and must be handed from one protein factor to another during its travel. Experimental analyses of diverse organisms uncovered proteins factors involved in tRNA import. All these factors had previously identified functions and are therefore described as multifunctional proteins. In each system, the tRNA import machineries seem to have distinctive features although a closer view suggests that common concepts are shared. This is illustrated by studies of mt-tRNA import systems described in three evolutionary divergent organisms (Figure 1).

The best-described import system is for cytosolic tRNA^Lys(CUU)^ in yeast mitochondria. In this system, the imported tRNA^Lys^ previously charged by the cytosolic lysyl-tRNA synthetase (LysRS) is specifically recognized and targeted to the mitochondrial surface by Eno2p (a glycolytic enolase). At the mt-surface, the tRNA is loaded on pre-MSK1 (the precursor of the mitochondrial lysyl-tRNA synthetase) that is synthesized at the mt-surface and enables the co-import of the tRNA via the protein import machinery while Eno2p is incorporated in the glycolytic complex associated to the mt-surface [57,69,70]. It is not yet clear how the translocation step is achieved but since pre-MSK1 can only charge the mt-encoded tRNA^Lys(UUU)^ and not the tRNA^Lys(CUU)^, the two molecules must be dissociated inside mitochondria to accomplish their respective function [58] (Figure 1a). It is noteworthy that components of the ubiquitin/26S proteasome system, Rpn13, Rpn8, and Doa1 (ubiquitin binding receptors), interact with the tRNA^Lys^ and the pre-MSK1 and might probably act in a regulatory way [71]. In land plants, *in vitro* analysis and biochemical approaches identified the Voltage Dependent Anion Channel (VDAC) as the main translocation channel through the outer mitochondrial membrane. However, proteins from the protein import machinery, *i.e.*, TOM20 and TOM40, are also involved in tRNA import and probably act as tRNA import receptors [72]. *In vitro*, tRNAs can enter mitochondria without any added protein factors [34,36,72] whereas *in vivo*, aminoacyl-tRNA synthetases (aaRSs) are required, although their exact role in import has to be characterized [73]. A protein shuttle system allowing to import any kind of RNA into isolated plant mitochondria showed that the adjunction of a nucleic acid binding protein improves tRNA import rates *in vitro* [74]. This suggests that cytosolic protein carriers, e.g., aaRSs, might be involved in tRNA import in plant mitochondria *in vivo* (Figure 1b). In *Leishmania tropica*, a system involving an inner mt-membrane RNA import complex was proposed but will not be discussed here as it is controversial and has raised editorial concerns [75]. Finally, tRNA import was described in *T. brucei*. In this organism, all mt-tRNAs are imported with the exception of the cytosol-specific tRNA^Met-i^ and tRNA^Sec^. *In vivo* and *vitro* analyses showed that imported tRNAs interact with the cytosolic elongation factor eEF1a indicating that this factor is implicated in the targeting of imported tRNAs [60,61]. An *in vivo* system showed that, contrary to plants, mitochondrial VDAC proteins are not required for import and that two mt-membrane proteins, *i.e.*, Tb11.01.4590 and Tb09.v1.0420, are part of the import machinery in the inner membrane [76,77]. In addition, the Tim17 and Hsp70 proteins belonging to the protein import machinery of the inner membrane were shown to be necessary for tRNA import into mitochondria [78]. A nine-subunit complex containing the Tim17, Hsp70, Hsp60 and Hsp20 proteins belonging to the protein import machinery were pulled-down with Tb11.01.4590, suggesting that mt-tRNA and protein import machineries in *T. brucei* share protein components. Alternatively, the two systems may constitute a common translocon but this remains to be established [77] (Figure 1c).

Beyond imported tRNAs, in many instances as discussed above, a set of tRNA genes remains encoded in mt-genomes of most eukaryote phyla. These tRNAs differ in many ways from cytosolic imported tRNAs. Their characterization and the identification of specific features started even before complete sequences of mitochondrial genomes became available.

## 3. Remarkable Structural Features of Mitochondrial tRNAs

### 3.1. Sequences and Secondary Structure

Among the first mt-tRNAs sequenced at the RNA level were mt-tRNAs from *Neurospora crassa* [79,80], *S. cerevisiae* [81,82] and *Phaseolus vulgaris* [83,84,85]. All these fungi and plant mt-tRNAs revealed primary and secondary structural features in line with canonical tRNAs, especially in terms of folding into cloverleaves, although peculiarities were noticed in terms of nt-content, low number of post-transcriptional modifications and changes in the content of conserved residues [86,87].

**Figure 2:**
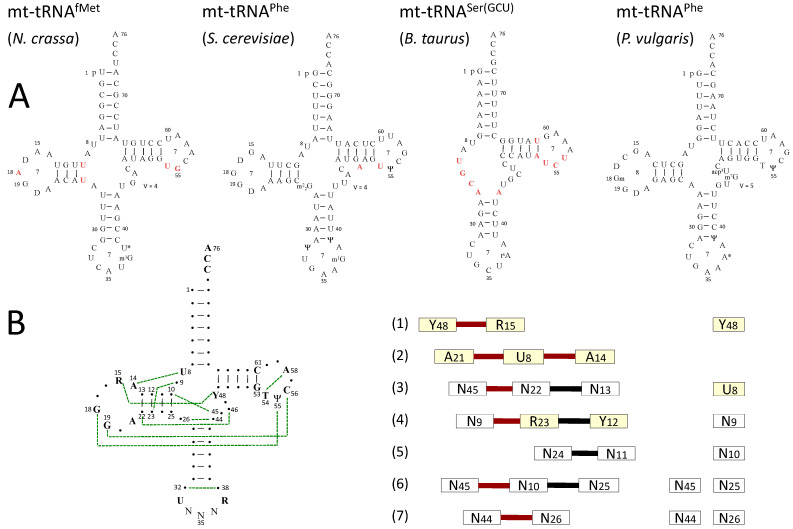
Typical sequences of mt-tRNAs displayed in cloverleaf-representation and comparison with the structural organization of cytosolic tRNAs. (**A**) Early examples of RNA sequences with post-transcriptional modifications shown in standard abbreviations [91] and deviations from the canonical cloverleaf coloured in red. These RNA sequences correspond to the first mt-tRNAs sequenced (*N. crassa* mt-tRNA^fMet^ and *S. cerevisiae* mt-tRNA^Phe^), the first bizarre mt-tRNA sequence (*Bos taurus* mt-tRNA^Ser(GCU)^) and the first sequenced plant mt-tRNA (*P. vulgaris* mt-tRNA^Phe^); (**B**) Canonical tRNA cloverleaf folding of cytosolic tRNAs and the core of the structure organized into seven base layers (including conserved and semi-conserved residues) that define the tRNA L-shape (R for purine, Y for pyrimidine, N for anticodon residues, dotted green lines for conserved tertiary pairings, red bars for atypical interaction present in three of the displayed sequences, but absent in bizarre tRNA^Ser^ and in some other mt-tRNAs and black bars for Watson-Crick interactions) (adapted from ref. [92]). For comparison, the simplified core within bizarre mt-tRNA^Ser(GCU)^ is shown. Note that the sequence of *P. vulgaris* mt-tRNA^Phe^ is of cytosolic-type, with conserved G18, G19, U55 and C56 needed for specific D/T-loop interaction.

The big surprise came from the sequences of the bovine and human mt-tRNA^Ser(GCU)^ isoacceptors that lack the complete D-arm [88,89], a feature conserved in all mammalian mt-genomes sequenced to date that encompass 22 sets of tRNA genes, namely two isoacceptors for tRNA^Ser^ and tRNA^Leu^ and one for each of the 18 remaining specificities. Beside this peculiarity, mammalian mt-tRNAs show a panel of other abnormalities ranging from mild to strong, including strong bias in nt-content for the 14 tRNAs encoded by the light DNA strand (A, U and C-rich tRNAs) and consequently a low number of G–C pairs in stem domains, large variability in D-loop and T-loop size (even for tRNAs of a same specificity but from different organisms) and absence in these loops of the conserved and semi-conserved signature motifs [90]. Figure 2 displays examples of RNA sequences of early investigated mt-tRNAs from lower fungi, plants and mammals and show how they deviate from cytosolic tRNAs.

An even bigger surprise came from the analysis of the mt-genomes from nematode worms indicating a systematic absence of either the D- or T-arms in several tRNAs (e.g., in *Ascaris suum* mt-tRNA^Asp^ missing the T-arm and tRNA^Ser(UCU)^ with short T-arm and without D-arm [93]). Furthermore, exploration of mt-genomes from acari, arachnids and nematodes revealed other large degenerations with extremely short and truncated tRNAs that can lack both D- and T-arms and have aberrant acceptor stems [94], as in acariform mites (e.g., from the genera *Dermatophagoites*, *Panonychus*, *Walchia* [92] or *Demodex* [95]) and in the nematode *Enoplea* group (e.g., in *Mermithidia* that are arthropod parasites, such the mosquito parasite *Romanomermis culicivorax* [96] and several spider mite mt-tRNAs from the genus *Tetranychus* [97]). These armless tRNAs are likely functional since *in vivo* transcription and 3'- and 5'-processing occur as demonstrated for several minimalist *R. culicivorax* mt-tRNAs [96], although explicit proof of aminoacylation or activity in translation is lacking (except for mammalian D-armless mt-tRNA^Ser(UCU)^ [98]).

### 3.2. Post-Transcriptional Modifications in mt-tRNAs

**Figure 3:**
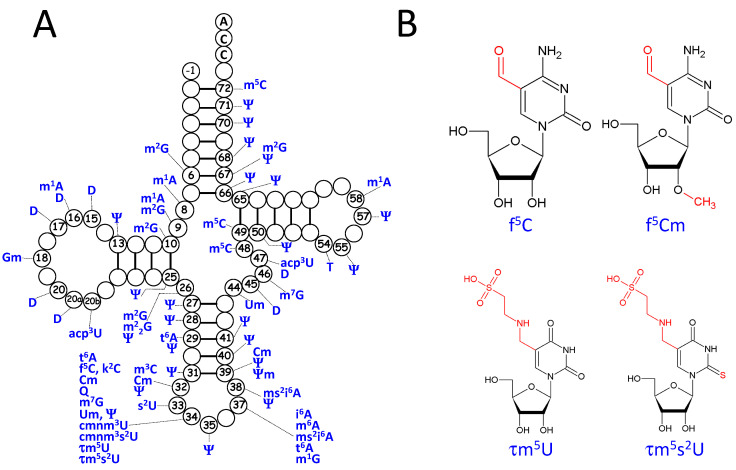
Distribution of post-transcriptional modifications in mt-tRNAs. (**A**) Localization of modifications in a tRNA cloverleaf (more details in Supplementary Table S1); (**B**) Structure of mitochondria-specific f^5^C and τm^5^U and their derivatives. Modifications are given in standard abbreviations [91]. The cloverleaf shows position −1, since some mt-tRNAs with His identity have a nucleotide at that position.

Post-transcriptional modifications are essential for tRNA structure and function [99,100]. However, knowledge in the mt-tRNA field is incomplete, due to difficulties in tRNA purification, sequencing and identification of the modified nucleosides. Indeed, only 123 sequences representing 22 species are known to date. Nonetheless, complete data are available for the 22 isolated mt-tRNAs from the liver of *Bos taurus* where mass spectrometry identified 15 types of modifications at 118 positions representing 7.5% of all beef liver mt-tRNA bases [101]. Compiled results are shown in Figure 3.

Altogether, 28 modified nucleosides scattered over 46 positions have been identified to date in mt-tRNAs. They include 15 out of 18 modifications from the so-called “Universal modifications” present in the three domains of life (only m^3^U, ac^4^C and m^6^_6_A are yet not found) [102] and mitochondria-specific residues at the wobble position 34, namely 5-formylcytidine (f^5^C and its methylated ribose f^5^Cm derivative) [103,104], four hypermodified U derivatives with notably 5-taurinomethyl-uridine (τm^5^U and its thiolated derivative τm^5^s^2^U) [105] and likely lysidine (k^2^C) [106]. Most of these modifications have been identified in mt-tRNAs isolated from echinoderms and mammals (~56%). In comparison with cytosolic tRNAs, the general trend is less diversity and a reduced number of modified residues in mt-tRNAs (e.g., in *Rattus norvegicus*, 3 nts in mt-tRNA^Asp^ compared to 8 in cytosolic tRNA^Asp^). Thus, the D-armless mt-tRNA^Ser(GCU)^ from the hamster *Mesocricetus auratus* [107] and a mt-tRNA^Gly^ isoacceptor from the primitive metazoan *Halocynthia roretzi* [108] are poor in modifications with a single modified base (t^6^A37) and two types of modifications (m^1^A and Ψ), respectively. Noteworthy, however, the *H. roretzi* mt-tRNA^Gly^ contains 4 Ψ's, what is a general trend in mt-tRNAs that are rich in Ψ  and Ψm residues, notably in stem regions (Supplementary Table S1). This feature could play a role in the structural plasticity of mt-tRNAs (see below). Furthermore, modifications in anticodon loops are often less sophisticated in mt-tRNAs than in their cytosolic tRNA counterparts, e.g., Q in mt-tRNA^Asp^ can be additionally modified to mannosyl-Q in cytololic vertebrate tRNA^Asp^. Finally, several modifications are of bacterial-type (e.g., m^6^t^6^A, cmnm^5^U and their s^2^ derivatives), consistent with the bacterial origin of mitochondria.

Modified residues act directly on the structure of mt-tRNAs and consequently their functions. Thus, Gm, Ψm and Cm residues enhance their chemical stability due to the methyl group on the 2' oxygen of ribose that prevent hydrolysis. Likewise, methyl or other chemical groups attached on Watson-Crick (WC) positions of bases (m^1^G, m^2^_2_G and acp^3^U) could prevent formation of false WC pairings detrimental to cloverleaf folding. In this view, the methyl group at m^1^A9 in human mt-tRNA^Lys^ has a key role in the folding process of this tRNA, since it prohibits a WC pairing between A9 and U64 from the T-stem that would trigger formation of hairpin instead of a cloverleaf fold [108,109,110]. On the other hand, and amply demonstrated for cytosolic tRNAs, modifications enhance the thermal stability of mt-tRNAs as illustrated by the melting profiles of the two beef mt-tRNA^Ser^ isoacceptors in their modified and unmodified versions, with a decrease of up to 6 °C for the transcript with almost normal cloverleaf [111]. Note that mt-tRNAs are globally unstable, since their melting temperatures in the range of 50–60 °C are significantly lower than those of cytosolic tRNAs (e.g., 76 °C for yeast tRNA^Phe^) [111]. In terms of functionality, modifications can be essential for correct codon reading in the peculiar mt-decoding systems [98]. This concerns modifications in anticodon loops, notably the mitochondria-specific formylated- and taurino-pyrimidines at the wobble position 34 of several mammalian mt-tRNAs [101].

In conclusion, knowledge about post-transcriptional modifications is essential to comprehend mt-tRNAs, but it is largely incomplete as it is biased for particular specificities and phyla. Thus, except for the complete sequence and modification landscape of *B. taurus* mt-tRNAs, sequence spaces for individual amino acid identities are poorly documented, with mt-tRNA^Leu^ sequences being the most represented (15-fold in eight species), while those with Ala, Cys, Gln and Pro identity are only represented two times each for one or two tRNAs. This is detrimental for the comprehensive understanding of mt-tRNA aminoacylation (see below). However, data on tRNA modification is expected to increase rapidly with the availability of novel technologies for tRNA purification and detection of modified nucleosides at micro and even nanoscales.

### 3.3. Three-Dimensional Structure of Free and Ligand-Bound mt-tRNAs

#### 3.3.1. Free mt-tRNAs

While most plant and fungal mt-tRNAs, as well as those from unicellular protozoans (e.g., *Tetrahymena*, *Paramecia*, *Plasmodia* species) share structural features with cytosolic tRNAs, metazoan mt-tRNAs frequently show structural features, often referred as bizarre. Still, they have to function in ribosome-mediated protein synthesis, implying conservation of an L-shaped architecture similar to that of cytosolic tRNAs. Rules based on sequence analysis, canonical tRNA conformation and structural compensations have been proposed to account for the L-shaped architecture of bizarre mt-tRNAs [112,113,114]. In canonical L-shaped tRNAs, the architecture is held together by a network of tertiary interactions divided in two domains (a compact core of seven base layers and a smaller D/T-loop interaction domain) comprising conserved and semi-conserved tRNA residues [92]. It is anticipated that similar or simplified networks account for the 3D structure of mt-tRNAs (Figure 2).

Since crystallographic data are lacking, explicit knowledge on the 3D structures of mt-tRNAs relies on solution data and modelling based on the crystal structures of cytosolic yeast tRNA^Phe^ and tRNA^Asp^. An overall L-shape was revealed by transient electric birefringence measurements on *H. sapiens* mt-tRNA^Lys^ [115] and *B. taurus* mt-tRNA^Ser(GCU)^ [116] with inter-stem angles, respectively of ~140° and ~120°, much larger than in the canonical yeast tRNAs. Note that the crystal structure of an archaeal tRNA^Pyl^, with sequence features close to mt-tRNAs, represents a tempting model of the 3D structure of mammalian mt-tRNA^Ser(UGA)^ [117,118]. On the other hand, an L-shaped model of beef and human mt-tRNA^Ser(GCU)^ (missing the D-arm) could be drawn based on an alternative network of tertiary interactions [113] and NMR identification of the predicted base pairs in helical regions [119]. Detailed structure probing on more classical mt-tRNAs with 4-arm-cloverleaves, notably *B. taurus* tRNA^Phe^ and *H. sapiens* mt-tRNA^Asp^, allowed to decipher their 3D interaction networks and to build L-shaped models [98,120]. For the shortest known mt-tRNAs (~40–50 nts) in which the missing D- and T-arms are replaced by short connectors joining the acceptor and anticodon arms, it was hypothesized that they gain flexibility for global L-bending through the connectors. This bended architecture is supported by the aminoacylation capacity of an engineered yeast tRNA^Asp^ in which the acceptor and anticodon helices are joined by two connectors [121]. Altogether, most mt-tRNAs are characterized by structural plasticity as reflected by high thermal instability [98] and particular flexibility in the connectors joining the acceptor with the anticodon region.

The determination of NMR and X-ray structures of anticodon-stem-loop (ASL) domains of mt-tRNA^Met(CAU)^ containing the f^5^C modifications at the functionally important position 34 was rewarding [122,123,124]. In short, the f^5^C modification contributes to the conformation of the anticodon domain and its ability to decode the AUA and AUG codons as Met in translational initiation and elongation [122] and to expand codon recognition from AUG to the synonymous AUU, AUC and AUA codons in the peculiar mt-decoding process [123]. Furthermore, its visualization by X-ray crystallography on the ribosome when bound to 6 nt long mRNA with AUA and AAA codons reveals that recognition of both A and G at the third position of the codons occurs in a canonical WC geometry. This is accompanied by a modification-dependent shift in the tautomeric equilibrium toward the rare imino-oxo tautomer of cytidine that stabilizes the f^5^C34–A base pair geometry with two H-bonds [124].

Given their structural diversity, mt-tRNAs can be ranked in five groups [92] (Table 2), characterized by roughly conserved distances between anticodon and terminal A76 as required for protein synthesis on the ribosome. The first group gathers tRNAs of quasi-canonical 3D structures from lower eukaryotes (e.g., fungi [125] and protozoans [126]) and plants [127] with standard core and D/T-loop interactions. The four other groups include the mt-tRNAs missing the canonical D/T-loop interactions and showing a progressively simplified core domain. Thus, tRNAs from the second group are quasi-canonical, but have lost conserved residues in the D- and T-loop, so that the contact between these loops will be perturbed (e.g., *H. sapiens* mt-tRNA^Asp^ and mt-tRNA^Phe^), and in addition can have an atypical D-arm (e.g., *T. pyriformis* mt-tRNA^Met^ [128]). In the third group, T-armless tRNAs are found, but with otherwise a conserved anticodon branch and a quasi-standard core (represented by *C. elegans* mt-tRNA^Asp^). The fourth and fifth groups comprise tRNAs with the most perturbed sequences. They are represented by *B. taurus* mt-tRNA^Ser(GCU)^ (Group 4) and minimalist tRNAs of less than 50 nts, such as *W. hayashii* mt-tRNA^Ala^ and *R. culicivorax* mt-tRNA^Arg^ (Group 5). Here, the changes in the core organization are profound, especially for tRNAs deprived of both D- and T-arms. Furthermore, the putative acceptor stem can be aberrant with a short size and a frequent absence of WC-pairings, as in arachnid mt-tRNAs [129,130].

**Table 2:** The five structural groups of mt-tRNAs. The classification is based on the analysis of tRNA sequences deduced from gene sequences and comparison with cytosolic tRNA sequences, in particular for the content of conserved and semi-conserved nucleotides. * The structural distinction within mt-tRNAs in alveolates and amoebas is not well known since sequence data from these phyla are scarce (e.g., few data on *Paramecia*, *Plasmodia*, *Tetrahymena*, *Acanthamoeba* species); ** Canonical loops: D-loops with G18G19 and size of 8 to 9 nts; T-loops with U54U55C56 (except in initiator tRNAs) and conserved size of 7 nts; *** Atypical loops of variable size: D-loops of 5–9 nts and T-loops of 6–7 nts (due to partial or total non-conservation of G18, G19, U55 and C56 that govern the D/T-loop interaction); **** D-stem can be restricted to 3 bp in some species. VR for Variable region, including putative helical domains in some mt-tRNA^Leu^, tRNA^Ser^ and tRNA^Tyr^ species. In Group 5, the size of the acceptor and anticodon helices deviate from the canonical organization (see Figure 4). Abbreviated names of eukaryal organisms: *Ath*, *Arabidopsis thaliana*; *Asu*, *Ascaris suum*; *Cel*, *Caenorhabditis elegans*; *Dno*, *Dasypusnovemcincus*; *Dya*, *Drosophila yakuba*; *Hsa*, *Homo sapiens*; *Mpo*, *Marchantia polymorpha*; *Pca*, *Pichia canadensis*; *Pvi*, *Phoca vitulina*; *Pvu*, *Phaseolus vulgaris*; *Sce*, *Saccharomyces cerevisiae*; *Rcu*, *Romanomermis culicivorax*; *Wha*, *Walchia hayashii*.

tRNA Groups	Group 1	Group 2	Group 3	Group 4	Group 5
Eukaryote groups	Alveolates & Amoebas * Fungi Plants	Alveolates & Amoebas * some Fungi & Plants Metazoans	Metazoans (e.g., nematodes, arachnids & some bryozoan species)	Metazoans (some insect & bryozoan species & mammals)	Metazoans (acaria & some nematodes)
Representative tRNAs	*Ath* mt-Asp *Sce* mt-Glu *Pca* mt-Leu^UAA^ *Pvu* mt-Phe	*Mpo* mt-Ala *Hsa* mt-Asp *Pca* mt-Cys *Hsa* mt-Phe	*Asu* mt-Ala *Asu* mt-Asn *Cel* mt-Asp *Asu* mt-Thr	*Dno* mt-Cys *Bta* mt-Ser^GCU^ *Dya* mt-Ser^GCU^ *Pvi* mt-Ser^GCU^	*Wha* mt-Ala *Rcu* mt-Arg *Rcu* mt-Ile *Rcu* mt-Thr
Acceptor arm	canonical (stem: 7 bp)	canonical (stem: 7 bp)	quasi-canonical (stem: 4–7 bp)	canonical (stem: 7 bp)	atypical (stem: 4–5 bp)
Anticodon arm	canonical (stem: 5 bp & loop: 7 nts)	canonical (stem: 5 bp & loop: 7 nts)	canonical (stem: 5 bp & loop: 7 nts)	canonical (stem: 5 bp & loop: 7 nts)	atypical (stem: 5–9 bp)
D- and T-arms	canonical **	atypical ***	atypical T-armless (atypical D-loops of 5–7 nts)	Atypical D-armless (atypical T-loops of 6–7 nts)	– D-/T-armless
T-arm	stem: 5 bp loop: 7 nts	stem: 4–5 bp loop: 6–7 nts	–	stem: 4–5 bp	–
D-arm	stem: 4 bp loop: 8–9 nts	stem: 4 bp ****	stem: 4 bp	–	–
L1 connector (size & characteristics)	19–20 nts (with D-arm)	16–20 nts (with D-arm)	16–18 nts (with D-arm)	5–12 nts	3–11 nts
L2 connector (size & characteristics)	21–30 nts (with VR & T-arm)	19–22 nts (with VR & T-arm)	6–7 nts	20–21 nts	5–14 nts
core organization in stacked base layers	7 layers (canonical)	7 layers (quasi-canonical)	7 layers (atypical)	6 layers (atypical)	1 layer (atypical)

Interestingly, in the minimalist tRNAs of the fifth group, the only conserved features characterizing cytosolic tRNAs are the helical acceptor arm with a 3'-single stranded CCA terminus and an anticodon arm with a strictly conserved canonical 7 nts-anticodon loop. This agrees with the NMR structure of the ASL domain of human mt-tRNA^Met^ that shows a canonical loop conformation [123]. The sequences of most mt-anticodon loops are furthermore in line with the structural signature of canonical anticodon loops, that among others conserves isosteric non-WC base pairs at position 32–38 and allows the presence of Ψ at position 35 [131]. The presence of m^7^G34 in mollusc and starfish tRNA^Ser(GCU)^ and the frequent occurrence of Ψ at position 31 are intriguing. Such Ψ residues could enhance the thermodynamic stability of the anticodon loops, as proposed for a cytosolic tRNA [132].

**Figure 4:**
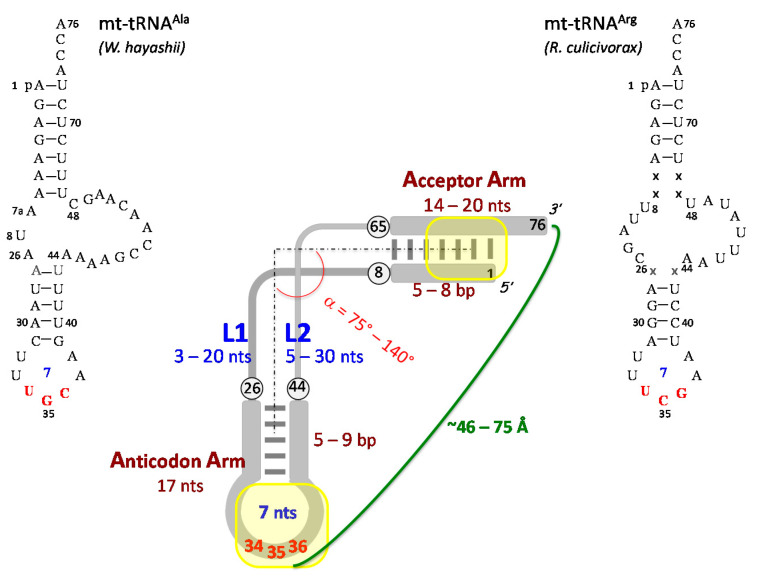
Generalized L-shaped structure of adapter tRNAs that highlights the minimalist structural requirements needed for tRNA participation in ribosome-mediated protein synthesis. Numbering of residues is as for canonical cytosolic tRNAs. As it occurs in cytosolic tRNAs but not conserved in mt-tRNAs, the generalized structure is displayed with an acceptor arm of seven base pairs (bp) and an anticodon arm of five bp (deviations in bp content are indicated). Localizations of major identity determinants at the distal extremities of the structure are shown. The sequence of two minimalist tRNAs restricted to the acceptor and anticodon arms joined by L1 and L2 connectors (*Walchia hayashii* mt-tRNA^Ala^ and *R. culicivorax* tRNA^Arg^) are in line with this generalized tRNA structure. Note that the minimalist tRNA^Arg^ (44 nts) is to date the shortest characterized tRNA [96]. Base pair content, missing position (x), and length of connectors’ sequences are indicated (in correspondence with sequence analysis mentioned in the text and Table 2).

Taking into account these facts, a generalized structure of tRNAs can be drawn, based on conservation of the anticodon arm and size and sequence features of the two connectors joining the acceptor and anticodon helices, namely L1 (nt 8–26) and L2 (nt 44–65) (Figure 4). It is notable that the seven base-layers that constitute the canonical core include exclusively residues from the connectors. This generalized structure accounts for most structure function relationships in mt-tRNAs participating in ribosome-dependent protein synthesis.

#### 3.3.2. mt-tRNAs Bound to aaRSs

Direct structural data on the binding of mt-tRNAs to mt-aaRSs are lacking. However, the crystal structures of *H. sapiens* mt-PheRS in complex with *Thermus thermophilus* tRNA^Phe^ is known [133] and tRNA-docking models based on the crystal structures of cognate *B. taurus* mt-SerRS and *H. sapiens* mt-AspRS were proposed [134,135].

The crystal structure of the heterologous mt-PheRS:tRNA^Phe^ complex provides useful information on mt-tRNA^Phe^ binding, since bacterial *T. thermophilus* tRNA^Phe^ is efficiently charged by the small and monomeric human mt-PheRS (cytosolic PheRSs are large and heterodimeric) [133,136] despite sequence deviations with human mt-tRNA^Phe^ but conservation of most core residues [90]. Thus, bound tRNA^Phe^ retains a majority of the tertiary interactions defining the L-shape, both in the compact core (e.g., the Hoogsteen pair U8–A14 and the Levitt pair G15–C48) and in the D/T interaction domain (e.g., the cis WC pair G19–C56 and the Hoogsteen pair U54–A58). Like in the bacterial cytosolic complex [137], the tRNA spans over the PheRS from the acceptor end to the anticodon, but the number of contacts with the small monomeric mt-PheRS is half than with the large heterodimeric *T. thermophilus* PheRS (25 *versus* 57). This is achieved by alternate interaction modes, especially with the anticodon-binding domain, a simplified recognition of the accepting stem and a conformational flexibility of nts 44 and 45.

Concerning the Ser system [134], docking indicates an interaction of the acceptor end of tRNA with mt-SerRS and excludes contacts with the anticodon arm. Furthermore, conformational adaptation of the tRNA elbow region and importance of the T-loop for specific recognition of the peculiar mt-tRNA^Ser^ species are suggested.

The situation is quite different in the Asp system [135]. Based on a structural model of human mt-tRNA^Asp^ [120] and the crystal structure of human mt-AspRS, it could be shown that the L-shaped tRNA spans over the enzyme from the inside of the L via specific contacts with identity elements from the anticodon but not with discriminator determinant G73 [135].

#### 3.3.3. mt-tRNAs Bound to Other Macromolecules and Macromolecular Assemblies

Beside mt-aaRSs, mt-tRNAs have to interact with numerous factors for tRNA maturation and translation itself. To date, however, the related structural biology is completely missing, except for the docking model of *Arabidopsis* mt-tRNA^Cys^ with PRORP1, a protein-only RNase P enzyme [138] (see below) and for the binding of the mt-ASL^Met(CAU)^ in the A-site of the 30S subunit of the ribosome from *T. thermophilus* [124] (see above). Mitoribosomes, however, are quite different and have undergone massive structural changes throughout their evolution [139]. These changes are explicitly seen in the 3D structures of the large subunit of the porcine [140] and yeast [141] mitoribosomes determined by cryo-EM, respectively at 4.9 and 3.2 Å resolution. Their remodelled architecture compared to the cytosolic counterparts is likely required for the exclusive synthesis of the hydrophobic mt-proteins and, in the case of the mammalian mitoribosome, for the recognition of the atypical conformations of mt-tRNAs, e.g., for the adjustment of the distance between the 3'-terminus and the anticodon consistent with that of usual tRNAs [119].

## 4. Biogenesis of Functional Mitochondrial tRNAs

### 4.1. Transcription

The organization of mt-genomes varies greatly across phyla. For instance, in metazoans and fungi, the mt-genome is small and compact (e.g., 16 kbp in human [142]), intergenic regions are almost missing and transcription is initiated from few promoters. In these genomes the observation that mRNA genes can be strictly delimited by tRNA genes led to the establishment of the tRNA punctuation model where the maturation of both tRNAs and mRNAs from long polycistronic transcripts is achieved by RNase P and RNase Z, the two endonucleases responsible for pre-tRNA 5'- and 3'-maturation, respectively [143]. tRNA punctuation is best exemplified by the human mt-genome where it accounts for nearly all RNA maturation events. Still, exceptions to this model have been documented in opisthokonts (the eukaryote group including the animal and fungi) [144]. In other eukaryote groups, mt-genomes have a very different organization. For example, in land plants, mt-genomes are much larger (e.g., 367 kbp in *Arabidopsis* [145]) and tRNA punctuation does not occur, although tRNA genes are sometimes clustered into operons. Transcription is initiated at multiple promoters and tRNA 3'-maturation requires the concerted action of both exonucleases and endonucleases similar to bacteria, the final steps of tRNA termini removal being performed by RNase P and RNase Z [14].

**Figure 5:**
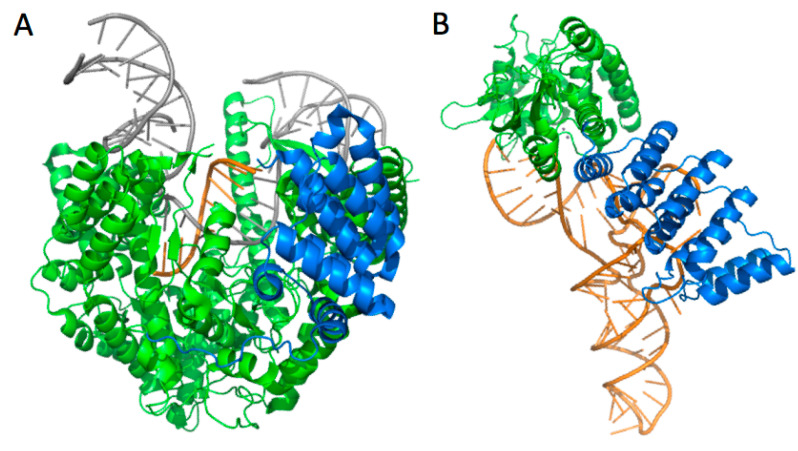
Many factors required for mitochondrial gene expression, *i.e.*, for the biogenesis of tRNA belong to the super-family of helical repeats modular proteins [170]. In particular, key factors for tRNA biogenesis in mitochondria belong to the PPR family. (**A**) Experimental structure of human mitochondrial RNA polymerase elongation complex and (**B**) model of *Arabidopsis* mitochondrial RNase P (AtPRORP1) in complex with mitochondrial tRNA^Cys^, with PPR domains highlighted in blue. RNA molecules are shown in orange and DNA in grey. The functions of the respective PPR domains are related to the exit of newly transcribed RNA molecules [147] and to the specific recognition of tRNA precursors [138,153].

While genomic organization is very divergent, the nature of enzymes responsible for the transcription of mt-tRNA is comparatively more conserved across phyla. In yeast and human, mt-RNA polymerases are nuclear-encoded single-subunit DNA-dependent RNA polymerases distantly related to bacteriophage T7 RNA polymerases [146]. Although, bacteriophage and mt-enzymes share conserved mechanisms for substrate binding and nucleotide incorporation, they also show strong mechanistic differences [147]. Interestingly, these enzymes contain pentatricopeptide repeat (PPR) domains, in agreement with recent studies that find a prevalence of PPR proteins for gene expression processes in mitochondria [148] (Figure 5). In human mt-RNA polymerase, PPR motifs are involved in the exit of newly synthesized RNAs [147]. In contrast, although plant mitochondria also use T7 related RNA polymerases [149], these enzymes paradoxically do not contain any PPR domain, in contradiction with the massive occurrence of PPR proteins in plants as compared to animals (there is e.g., 80 times more PPR genes in *Arabidopsis* than in human) [150]. Beyond the wide occurrence of phage-type polymerases, some groups such as jakobides seem to have retained bacterial-like mt-RNA polymerases. For example, in *Reclinomonas americana*, the 69 kbp mt-genome encodes four subunits of a eubacterial-type RNA polymerase. This is in agreement with the ancestral nature of *R. americana* mt-DNA which is the most eubacteria-like mt-genome identified so far [151].

### 4.2. Maturation of tRNA 5'- and 3'-Termini

#### 4.2.1. Maturation at 5'-Terminus

After transcription, tRNA precursors leader and trailer sequences are removed by endonuclease activities. 5'-Maturation is performed by RNase P first characterized in several bacterial species where it was found to be implemented by ribonucleoproteins (RNP) comprising a ribozyme [152]. In this context, and because of the prokaryotic origin of mitochondria, it was assumed that mt-RNase P would universally occur as RNPs [153]. This assumption was supported by the identification of the corresponding RNA subunit (P-RNA) genes in yeast mitochondria [154], in glaucophyte cyanelles [155] and in jakobides, including *R. americana* mitochondria [151,156]. In particular, within the fungal lineage, most of the known P-RNA genes are found in the saccharomycete lineages from the ascomycete group. These P-RNA structural features are highly reduced, *i.e.*, they essentially contain helix P4, the activity centre of the ribozyme and P1 that pairs the 5'- and 3'-termini [154]. The protein composition of mt-RNP RNase P is overall poorly characterized but seems to be distinct from bacterial or nuclear RNP RNase P protein composition [157].

While RNP RNases P were identified in different eukaryote groups, the analysis of genomic sequences revealed that several eukaryote groups, such as kinetoplastids or land plants, do not encode a recognizable P-RNA or RNase P-specific proteins, neither in the organellar nor in the nuclear genome [157]. This suggested that another type of RNase P must exist in eukaryotes. This hypothesis was supported by earlier biochemical analyses performed on spinach chloroplasts and human mitochondria [153], but only definitively demonstrated with the molecular characterization of RNase P enzymes in human mitochondria [158] and plant organelles [159]. This other type of RNase P enzyme, termed PRORP for “protein-only RNase P”, comprises a PPR RNA binding domain as well as a NYN catalytic domain, and is devoid of any RNA component (Figure 5). Interestingly, while in *Arabidopsis* PRORP functions as a single subunit enzyme, in human PRORP requires two additional subunits for the 5' cleavage of human mt-tRNA precursors. This is probably due to the non-canonical nature of human mt-tRNAs [144]. Beyond *Arabidopsis* and human, mitochondrial PRORP were also characterized in trypanosome [160] and in the moss *Physcomitrella patens* [161]. However, contrary to initial beliefs, PRORP proteins are not specific to mitochondria (and organelles in general). They also frequently occur as nuclear RNase P enzymes, *i.e.*, up to now characterized in *Arabidopsis* [162], trypanosome [160] and *P. patens* [161]. PRORP genes are not of prokaryote origin and have evolved from eukaryote specific nuclear genes [153]. In some groups, e.g., land plants PRORP enzymes have entirely replaced RNPs for RNase P activity [162]. Interestingly, although PRORP and RNP RNase P employ different catalytic strategies [163], it seems that PRORP holds a substrate recognition mechanism similar to that of RNP RNase P [138].

#### 4.2.2. Maturation at 3'-Terminus

Then, the 3'-maturation of tRNAs involves another endonuclease called RNase Z. Contrary to RNase P, the nature of RNase Z is much more conserved, with all enzymes belonging to the same family. RNase Z genes are part of the super-group of β-lactamases. They are related to metallo-hydrolases and characterized by a conserved α–β/β–α fold. They also harbour a conserved metal ion-coordinating site often binding zinc and possess an additional flexible arm called exosite involved in the binding of pre-tRNAs [144,164]. RNase Z enzymes occur in two major forms, a shorter version called RNase Z^S^ is found in the three domains of life, while another version, called RNase Z^L^, approximately twice as large as the former, only occurs in eukaryotes. Interestingly, in mitochondria RNases Z always correspond to the longer eukaryote-specific form. In human, two RNase Z genes are present. A shorter RNase Z^S^ is found in the cytosol, while ELAC2 (elaC ribonuclease Z 2) a protein belonging to the RNase Z^L^ group is dual localized to mitochondria and the nucleus [165,166]. In *Arabidopsis*, four RNase Z proteins are encoded in the nucleus, among which one RNase Z^L^ is shared between nucleus and mitochondria, while another one is restricted to mitochondria [167].

Finally, a last critical step to obtain functional mt-tRNAs consists in the addition of the CCA 3' terminal group. While CCA is encoded in tRNA genes from prokaryotes such as *E. coli*, it is absent from nearly all eubacterial tRNA genes. Similarly, it is always absent from mitochondrial tRNA genes and has to be added post-transcriptionally by CCA-adding enzymes [168]. In this line, it is interesting to note that animal tRNA nucleotidyltransferases resemble eubacterial enzymes, thus suggesting that animal CCA adding enzymes might have been acquired from the endosymbiotic ancestor of mitochondria [169].

### 4.3. Functions and Mechanisms for tRNA Modifications and Editing

After or during the processing of transcript termini, pre-tRNAs undergo a series of modification and editing events in order to become functional. While the term RNA “processing” generally reflects changes affecting pieces of sequence through cutting and/or rejoining processes, the term “modification” describes biochemical processes resulting in the *in situ* formation or introduction of non-standard nucleotides, and as such, is mostly found in tRNAs. In contrast, the term RNA “editing”, while initially coined to describe RNA alterations that change the coded message in mRNA [171] is also used in a more general sense for tRNAs and includes all A, C, G or U sequence changes that take place at the RNA level other than splicing and polyadenylation that could in principle have been directly encoded by DNA [172].

#### 4.3.1. Mitochondrial tRNA Modifications

The general view on the function of tRNA modifications is that most of them enhance the formation or stabilize correct tRNA structures or prevent the formation of incorrect ones. As amply demonstrated for cytosolic tRNAs where tRNA modifications are essential for efficient and accurate anticodon/codon interactions [102], they play also a primordial role in mitochondrial translational accuracy, especially for decoding reassigned codons [98]. As an example, f^5^C at the wobble position 34 expands codon recognition from the traditional AUG to the non-traditional, synonymous codons AUU and AUC as well as AUA, in mitochondria [122,123]. Modifications can also be signals for the recognition by aaRSs, by the ribosome or by other unidentified partners. They might also serve as aminoacylation antideterminants. However, individual modification losses result most of the times in subtle molecular effects on the function of tRNAs and on translation as they can generally be compensated by other modifications [102,173]. Still, at the macroscopic level, modification defects can lead to severe disorders such as mitochondrial diseases [174]. In particular, MELAS and MERRF syndromes were connected to taurine τm^5^U and τm^5^s^2^U deficiencies at the anticodon wobble position [175,176,177].

In mitochondria, tRNA modifications and the enzymes responsible for these activities have essentially been characterized in animals and yeast. A recent comprehensive analysis performed on the bovine mt-tRNAs identified 15 types of modified nucleosides distributed over 7.5% of all mt-tRNA bases [101] (see above for details). Although post-transcriptional modifications in mt-tRNAs are less abundant than in cytosolic tRNAs, their biogenesis requires a large panel of specialized enzymes, some exclusively dedicated to functions in mitochondria. Among them, MTU1 in human and yeast, is the mt-tRNA specific 2-thiouridylase involved in the biogenesis of taurine-containing modified uridine τm^5^s^2^U at the anticodon wobble position [105,178]. To date, ~10 mt-tRNA modifying enzymes are functionally characterized in animals, although the analysis of genomes identifies many more candidate genes. The characterized enzymes include methyltransferases, methylthiotransferases, isopentenyltransferase and pseudouridine synthases [101]. Similarly, in plants, many putative tRNA modification enzymes are predicted, although experimental data on mt-enzymes are lacking [127]. Interestingly TRMT10C and SDR5C1 that are responsible for methylations at position 9 of several tRNAs in human mitochondria are also essential partners of PRORP for protein-only RNase P activity, thus illustrating how tRNA modifying enzymes can be involved in distinct activities as part of multifunctional complexes [158,179].

#### 4.3.2. Mitochondrial tRNA Editing

Similar to other mitochondrial encoded RNA species, mt-tRNAs can be affected by a diverse array of editing processes. These events are most of the time specific to given eukaryote phyla and mechanistically unrelated. In metazoan, despite the prevalence of tRNA punctuation, tRNA genes sometimes overlap (between 1 and 6 nts) and RNA editing is used to correct primary transcript processing products [180,181]. For example, in human mitochondria, tRNA^Tyr^ and tRNA^Cys^ present a one base overlap. The upstream tRNA^Tyr^ is released by an endonuclease activity independent from RNase P and the truncated tRNA^Tyr^ is repaired by an RNA editing activity that adds the missing nucleotide [182]. In other examples, several nucleotide exchanges in the first three nucleotides of the mt-tRNA acceptor stem take place in the amoeboid protozoans *Acanthamoeba* mt-tRNAs but also in lower fungi such as *Chytridiomyceta* [183,184]. In contrast, in a number of mt-tRNAs from *Myxomyceta* such as *Physarum polcephalum*, RNA editing consists in the insertion of C and U residues. The occurrence of partially edited sequences in tRNA precursors suggests that this type of editing might be a prerequisite for tRNA processing [185]. Nonetheless, the most frequent type of RNA editing described to date corresponds to C to U alterations due to the deamination of cytidines. For example, in marsupials, RNA editing alters the identity of mt-tRNAs as it corrects the GCC anticodon of tRNA^Asp^ to GUC [186]. In land plants, C to U RNA editing is prevalent as it affects hundreds of positions in the mitochondrial transcriptome [187,188]. Although less frequent than in mRNAs, this type of C to U editing also affects mt-tRNAs [189]. In several instances, it seems that tRNA editing is a prerequisite for tRNA processing. For example in several plants (but not in *Arabidopsis*) mt-tRNA^Phe^ is edited at position 4. This change that corrects a mismatch in the acceptor stem appears to be required for 5'-processing of tRNA precursors by RNase P [190]. It is very likely, but not demonstrated, that the machinery that performs tRNAs C to U editing is the same that edits mRNAs. It involves PPR proteins for the site-specific recognition of cytidines. More precisely, it implicates in some instances proteins of the PPR-DYW subgroup, which contain a domain resembling a cytidine deaminase and were predicted to perform the actual catalytic reaction of RNA editing [14,191].

Beyond the mitochondrial encoded tRNAs, editing processes were also reported for cytosolic tRNAs imported into mitochondria. For instance, it was shown in *L. tarentolae* that a specific C to U editing in the anticodon of the imported nonedited tRNA^Trp^ allows the decoding of mitochondrial UGA codons as tryptophans [192]. In contrast, in the moss *P. patens* a tRNA^Arg(ACG)^ isoacceptor encoded in mitochondria, thus not imported from the cytosol, is deaminated at position A34 to obtain an ICG anticodon [193]. Interestingly for protozoan tRNA^Trp^, import and editing have drastic structural consequences, since this tRNA is partially thiolated at universally conserved position 33 to s^2^U33 and O′-methylated at the ribose moiety of Ψ32 as well as on edited U34 and nonedited C34 [194]. The presence of s^2^U33 and the concentration of O′-methylated pyrimidines in the anticodon domain, likely is the prerequisite for proper decoding of the Trp codon by conferring conformation rigidity and chemical stability to the anticodon loop. In contrast, dethiolation of cmm^5^s^2^U within mitochondria at wobble position 34 of imported trypanosomatid tRNAs, as explicitly shown for *T. brucei* tRNA^Glu^, is an alternate editing mechanisms required to modulate decoding [195].

## 5. Aminoacylation of Mitochondrial tRNAs

### 5.1. Global Features of Mitochondrial tRNA Aminoacylation Systems

Aminoacylation of mt-tRNAs is catalyzed by imported nuclear encoded mt-aaRSs using the same type of two-step reactions than cytosolic tRNAs, namely formation of a transient aminoacyl-adenylate followed by the transfer of the activated amino acid to tRNA. Most mt-aaRSs are bacterial-like [196]. Exceptions are nuclear-encoded aaRSs that are active in both cytosol and mitochondria, as human GlyRS and LysRS [197] and mt-aaRSs lost from the nuclear genome in organisms where the homologous mt-tRNA encoded in the mt-genome has been lost, as in the ciliated protozoa *Nematostella vectensis* [198]. The nuclear-encoded mt-aaRSs have intricate phylogenetic origins with gene duplication and horizontal gene transfer from bacterial endosymbionts to eukaryotes. As a consequence, mt-aaRSs present structural idiosyncrasies when compared to their cytosolic homologues, the most dramatic occurring in mt-PheRSs that are monomeric [199,200] in sharp contrast with bacterial and other cytosolic PheRSs that are tetramers. This nuclear origin implies that their gene products are targeted towards the organelles. In plants, as documented for *A. thaliana*, the same bacterial-like gene product is dual-targeted by different *N*-terminal signatures for delivery in either mitochondria or chloroplasts [201,202]. It is noteworthy that mt-aaRSs seem to exhibit a reduced catalytic efficiency for tRNA aminoacylation as compared to that of the bacterial or cytosolic homologues, as explicitly shown for several yeast and mammalian mt-aaRSs [197,199,203].

Like in many bacteria, GlnRS is absent in mitochondria, so that glutaminyl-tRNA^Gln^ is formed by an indirect transamidation pathway where tRNA^Gln^ is first charged with glutamate by a nondiscriminating GluRS (aminoacylating both tRNA^Glu^ and tRNA^Gln^) and converted into glutaminyl-tRNA^Gln^ by tRNA-dependent amidotransferases. The bacterial pathway is well known [204,205] and occurs in mitochondria with imported glutamyl-tRNA^Gln^ amidotransferase (GAT) subunits as demonstrated in plant [206], yeast [207,208] and mammalian [209,210] mitochondria. In plants mt-tRNA^Gln^ is mt-encoded and the indirect pathway also occurs in chloroplasts and apicoplasts [206,211]. It is also noteworthy that the indirect pathway for asparginyl-tRNA^Asn^ synthesis [204], frequent in bacteria when AsnRS is missing, does not occur in mitochondria.

Mt-aaRSs are often able to aminoacylate both mitochondrial and bacterial tRNAs, but bacterial aaRSs do not or only weakly aminoacylate animal mt-tRNAs [212,213]. Likewise, mt-AspRS from *T. brucei* aspartylates both homologous cytosolic tRNA^Asp^ and mt-tRNA^Asp^, while cytosolic AspRS exclusively aspartylates cytosolic tRNA^Asp^ [214]. These behaviours are likely linked to the large sequence and structural relaxation of mt-tRNAs, itself a consequence of the high rate of mt-genome divergence due to rapid evolution in mitochondria [215]. For plant and fungal systems the situation is more intricate, since organellar tRNAs and aaRSs can have diverse origins and evolutionary histories [53] and consequently share characteristics of heterologous cytosolic aminoacylation systems. Specificity in such systems can be due to the delocalization of a critical aminoacylation identity element in the tRNA sequence or to structural peculiarities in tRNA or aaRS. This raises questions about the fidelity of tRNA aminoacylation in mitochondria and the identity of mt-tRNAs.

### 5.2. Aminoacylation Identity of mt-tRNAs

#### 5.2.1. General Considerations

The designation “tRNA identity” is often used in the tRNA literature, notably to describe the different types of tRNAs found in mitochondria [114]. This designation, however, can be misleading if it solely correlates with codon decoding. Given that the genetic code is defined by specific tRNA aminoacylation by aaRSs (since nonspecific aminoacylations can lead to false amino acid incorporations into proteins), tRNA identity should be defined by the determinants specifying aminoacylation [216]. This point is of particular importance for mt-tRNAs where peculiarities in the mt-genetic code [98] and RNA editing events [217] can affect codon decoding or modify the anticodon of tRNA, and consequently could be the cause of tRNA identity changes.

tRNA identity is discussed here in the light of aminoacylation for which specificity is accounted by identity rules. These rules rely on limited set of determinants within a given tRNA generally interacting with the cognate aaRSs and on less well-characterized antideterminants that prevent tRNA interactions with non-cognate aaRSs. Strength of individual determinants (or sets of determinants) is variable and is estimated by the loss of catalytic efficiency or the suppression ability upon their mutation. In most systems, determinants are concentrated in the anticodon loop (mainly in anticodons) and the acceptor stem, but some are found in the core region of tRNA. For a given identity, strong determinants occur mostly at both distal extremities of the tRNA, but for Ala identity they are restricted to the G3–U70 pair in the acceptor stem. Identity rules are rather well understood for cytosolic tRNAs [216,218], but remain elusive for mt-tRNAs.

Because all mt-tRNAs have a conserved anticodon and acceptor region (Figure 3), it is anticipated, as for cytosolic tRNAs, that elements from these extremities contribute to mt-identity. On the other hand, because of the huge structural variability of mt-tRNAs and their ranking in structural groups (Table 2), group-dependent idiosyncrasies are anticipated in mt-identity sets. However, limited information is available for identity rules in mt-systems and proofs of expectations are scarce, essentially because of the difficulty to run mutational analyses, as done for cytosolic tRNAs. The currently available data on Ala, Asp, Leu, Phe, Ser and Tyr identity covering different structural-types of mt-tRNAs are summarized below.

#### 5.2.2. Understanding mt-tRNA Identities for Aminoacylations Catalyzed by Class I aaRSs

**Leu identity**: Based on solution probing, it has been shown that the *in vitro* transcribed human mt-tRNA^Leu(UUR)^ (a Group 2 mt-tRNA, Table 2) does not fold into the expected cloverleaf, but is nevertheless aminoacylated by human mt-LeuRS despite a partially disordered structure with a floppy anticodon branch [219]. Contacts with mt-LeuRS occur via its amino acid acceptor stem, anticodon stem and D-loop, which is unprecedented in Leu aminoacylation systems [220]. This suggests an adaptation of the tRNA structure with LeuRS upon binding. A mutational analysis shows that the leucylation activity is dependent on two strong Leu identity determinants (A14 in the D-loop and A73 at the discriminator position) [219], suggesting that Leu identity obeys rules similar to those that apply in *E. coli* [218]. Two mutations at identity position 14 are correlated with severe human pathologies [221,222], but impact leucylation efficiency differently, *i.e.*, 300-fold for the A14U mutant and only 10-fold for the A14G mutant. Similarly to the weak impact on leucylation of the A14G mutation, three other pathology-related mutations (U20C, U40C, C72U) do not affect leucylation [219]. Thus, as also observed with other mt-tRNAs, decreased aminoacylation capacity is not the unique cause of pathologies [223].

**Tyr identity**: Identity of human mt-tRNA^Tyr^ (a Group 2 mt-tRNA) for specific tyrosylation results from an extreme case of mutual adaptation of TyrRS and tRNA. Indeed, this mt-TyrRS presents dual sequence features of eubacterial and archaeal TyrRSs, especially in the region containing amino acids recognizing the major N1–N72 Tyr identity pair. As a result, human mt-TyrRS has lost the capacity to discriminate between the G1–C72 pair typical of eubacterial and mt-tRNA^Tyr^ and the reverse C1–G72 pair present in archaeal and eukaryal tRNA^Tyr^. Sequence comparisons of mt-TyrRSs across phylogeny suggest that this behaviour is conserved among vertebrate mt-TyrRSs, so that Tyr identity of mt-tRNA^Tyr^ would essentially rely on the discriminator base A73, since its mutation into G73 abolishes tyrosylation [224,225]. This conclusion finds support in the crystal structure of the mt-TyrRS that presents features not seen in eubacterial TyrRSs, notably bulges at the enzyme surface and an idiosyncratic electrostatic surface potential. Furthermore, mutagenesis of the catalytic domain reveals the importance of Ser200 in line with an involvement of A73 rather than N1–N72 in Tyr identity [226].

**Trp identity**: Tryptophanylation of *Oryza sativa* mt-tRNA^Trp^ (a Group 1 mt-tRNA) by *Bacillus subtilis* and human TrpRSs show great changes in aminoacylation efficiency due to species-specific identity elements in the plant mt-tRNA^Trp^. These elements are similar to bacterial and eukaryotic Trp identity determinants, notably the discriminator base G73 and the two base pairs G1–U72 and U5–A68 in the acceptor stem. Altogether, this supports the hypothesis that mt-tRNA^Trp^ is of eubacterial origin [227].

#### 5.2.3. Understanding mt-tRNA Identities for Aminoacylations Catalyzed by Class II aaRSs

**Ser identity**: Identity of the two bovine mt-tRNA^Ser(GCU)^ and mt-tRNA^Ser(UGA)^ isoacceptors (respectively, Group 4 and Group 2 mt-tRNAs) soon attracted interest because these topologically distinct tRNAs are both recognized and serylated by a single mt-SerRS. Footprinting and kinetic studies show that the mt-SerRS recognizes specifically the T-loop of each isoacceptor. However, for mt-tRNA^Ser(UGA)^, the T-/D-loop interaction is further required for recognition, suggesting that mt-SerRS recognizes its two substrates by distinct mechanisms [228]. Crystallographic data combined with mutagenesis and docking studies, confirm the dual recognition mode and clearly show that the mt-SerRS recognizes the distinct shape of each tRNA^Ser^ isoacceptor by indirect readout involving recognition of the backbone of the acceptor helix and alternative interactions with the tRNA core [134]. This is in contrast with Ser identity in cytoplasmic tRNAs, e.g., in *E. coli* where the major Ser identity determinant is the long extra arm [229].

**Asp identity**: The unexpected finding was the loss in human mt-tRNA^Asp^ (a Group 2 mt-tRNA) of the major Asp identity determinant G73 [213]. This fact is explained by the crystallographic and biophysical properties of human mt-AspRS and by the thermodynamics of tRNA^Asp^:AspRS complex formation. Even though the 3D structure of the mt-AspRS is close to that of the *E. coli* homologue, it differentiates by an enlarged catalytic groove, a more electropositive surface and a strongly reduced thermal stability. Moreover, isothermal titration calorimetry shows a higher affinity of mt-AspRS for cognate tRNA than for noncognate tRNAs, but with different enthalpic and entropic contributions [135]. Furthermore, analysis of *in vitro* transcribed human mt-tRNA^Asp^ variants tested for their ability to be aspartylated by *E. coli* AspRS, reveals that full conversion into a molecule as active as *E. coli* tRNA^Asp^ cannot be achieved on the basis of the currently established tRNA/aaRS recognition rules. Indeed, transplantation of the full set of Asp identity elements and stabilization of the tRNA scaffold by restoration of the D/T-loop interactions, enables only a partial gain in aspartylation efficiency. The sequence context and high structural instability of mt-tRNA^Asp^ are additional features hindering optimal adaptation of the tRNA to the bacterial enzyme [205].

On the other hand, in marsupials, mt-tRNA^Asp^ is encoded with a glycine anticodon GCC and requires editing of this anticodon to create the aspartate GUC anticodon and thereby to acquire Asp identity (while keeping glycylation capacity). Thus, replacing an amino group with a keto group at position 35 in the anticodon, changes the identity of the tRNA and allows a single gene to encode two tRNAs [186].

**Ala identity**: This identity is of particular interest since the major G3–U70 determinant in the acceptor helix, while widely distributed in cytoplasmic tRNAs and few mt-tRNAs (e.g., in some fungi, nematode and plant mt-tRNA^Ala^) is absent (e.g., in *W. hayashii* mt-tRNA^Ala^, see Figure 4) or translocated to adjacent positions in many putative mt-tRNA^Ala^ molecules (e.g., in some arachnid and bryozoan mt-tRNAs [129,230]. Thus, *Drosophila melanogaster* mt-tRNA^Ala^ (a Group 2 tRNA) has a translocated G–U pair at the 2–71 positions. This translocated G2–U71 and the adjacent G3–C70 are the major determinants for recognition by the *Drosophila* mt-AlaRS. Additionally, G3–U70 serves as an antideterminant for *Drosophila* mt-AlaRS so that the mt-AlaRS cannot charge cytoplasmic tRNA^Ala^ [231]. These features seem to be conserved within insect Ala systems. Interestingly, in *C. elegans* where the G–U pair is preserved at the 3–70 position, the proximal nucleotide U72 blocks charging by *E. coli* AlaRS [232]. The absence of a G–U pair in the acceptor helix of putative mt-tRNA^Ala^ species (e.g., from *T. brucei*) remains unexplained.

In contrast, in plants, mt-tRNA^Ala^ (a Group 1 tRNA) is imported from the cytosol and requires the G3–U70 determinant for both aminoacylation and import. This was clearly demonstrated with *A. thaliana* tRNA^Ala^ that loses both alanylation capacity and ability to be imported when U70 is mutated to C70 [33]. The situation of mt-tRNA^Ala^ from the moss *Celleporella hyalina* [230] and from several arachnid orders [129] is particularly intriguing and questions the exact Ala identity rules in these organisms, since mt-genomes show absence of the major G3–U70 Ala determinant and suggest an aberrant acceptor helix.

**Phe identity**: First information came from studies with *S. cerevisiae* mt-PheRS (a conserved monomer in mitochondria, in contrast with the tetrameric α_2_β_2_ cytosolic PheRSs), an enzyme that efficiently aminoacylates yeast tRNA^Phe^ [233]. The phenylalanylation capacity of a large panel of yeast tRNA^Phe^ transcripts indicates that both monomeric and tetrameric PheRSs are sensitive to the same determinants, namely the GAA anticodon triplet, the last G1–C72 pair in the acceptor helix and discriminator A73. However, mt-PheRS seems less sensitive to the tertiary structure of tRNA than the cytoplasmic PheRS [233]. More recent information based on crystallography concern Phe identity of human mt-tRNA^Phe^ for recognition by the minimalist human mt-PheRS that consists solely of two structural domains [133]. This aaRS has a broad specificity since it aminoacylates efficiently tRNA^Phe^ transcripts of bacterial, eukaryal, chloroplastic and mitochondrial origin [133]. But, unlike *E. coli* PheRS that recognizes an identity set of 11 nts (from both extremities and the central core of L-shaped tRNA) [234], the human mt-PheRS interacts with a restricted set consisting primarily of the G1–C72 pair and the discriminator base A73, also proposed to contribute to tRNA^Phe^ identity in yeast mitochondria. Recognition of G34 from the anticodon requires huge conformational adaptations with a rearrangement of the anticodon-binding domain and repositioning of tRNA via long-range electrostatic interactions [133].

#### 5.2.4. Conserved Features and Peculiarities in mt-tRNA Aminoacylation Identity: An Overview

Despite recognition and aminoacylation specificities, mt-tRNAs from Group 1 (*i.e.*, most mt-tRNAs with canonical structure found in plants and fungi) seem to follow the “universal identity rules” of cytoplasmic tRNAs. In contrast, many peculiarities are observed within mt-tRNAs from the other groups (e.g., those with aberrant structure). The limited and partial results for these mt-tRNAs (only seven identities have been superficially investigated) suggest that mt-aaRSs recognize minimalist identity sets that can be restricted to the sole anticodon bases or elements from the acceptor branch. Additionally, as shown for Ser identity, conformational motifs in mt-tRNAs likely serve as major identity determinants. Such “conformational” determinants will certainly be essential for the activity of the bizarre mt-tRNAs from Groups 3, 4 and 5, for which aminoacylation capacity is still not explicitly demonstrated for most of them. Species-specific conformational flexibility of mt-tRNAs seems to be a common theme. The degree of flexibility, likely, could be modulated by post-transcriptional modifications. Thus, as discussed for cytosolic tRNAs [235], individual modified bases could act as positive determinants or negative antideterminants in mt-tRNAs, and moreover could contribute collectively to identity. Altogether, specific aminoacylation needed for correct protein synthesis in mitochondria is the result of intricate evolutionary mechanisms for adaptation of nuclear-encoded aaRSs to degenerate mt-encoded tRNAs. In support to this possibility, some of the modified bases localized in anticodon loops that are identity elements in cytosolic tRNAs (e.g., Ψ35 for Tyr identity or t^6^A37 for Ile identity) are found at similar positions in homologous tyrosine or isoleucine mt-tRNAs [235].

Most extreme peculiarities come from the increasing amount of sequence data of mt-genomes that suggest existence of armless mt-tRNAs or with aberrant acceptor stems and possibly without helical folding. Although the precise structure and the aminoacylation ability of most of these putative tRNAs remain unexplored, old data from literature support the possibility that tRNAs without helical acceptor stem can be aminoacylated. This concerns a fragment of cytosolic yeast tRNA^Phe^ with an excised 5'-quarter, that can be efficiently aminoacylated *in vitro* by yeast PheRS provided the m7 group of G46 is removed [236]. Also, a polyU (~30 U) with an attached 3'-CCA tail is aminoacylated by a mammalian LysRS [237]. In both cases, the tRNA-mimics has great conformational flexibility and contain the major Phe or Lys identity determinants from the anticodon [218]. In this context it is worth mentioning putative mt-tRNA^Phe^ and mt-tRNA^Lys^ molecules with aberrant acceptor ends in arachnides from the genus *Aphonopelma* and *Hypochilus* [129]. Interestingly, a putative mt-tRNA^Ala^ with single-stranded acceptor stem is predicted in *Hypochilus thorelli*, but not in *Aphonopelma* [129].

## 6. Conclusions and Perspectives

Although the requirement of mt-tRNAs for mt-translation is obvious, the underlying molecular and functional biology is diverse and only partly explored across the eukaryal world. Only a few phylogenetic groups, mostly isolated species, have been extensively studied and often only for certain aspects. Many conclusions on mt-tRNAs are based on bioinformatic exploration of mt-genomes without experimental validation of the actual structures and functions of tRNAs. Huge technical difficulties for preparing the required amounts of molecules or to conduct appropriate genetic experiments explain this lack of validations, especially for mt-tRNAs in unusual taxonomic niches. Still, it appears that mitochondria have evolved many independent features for all aspects of tRNA biology in their respective eukaryote phyla. Consequently, generalizations about overall tRNA structural and functional features in mitochondria are not easy to propose. Diversity has arisen with differences in evolution rates across eukaryotic phyla and with different genetic exchange processes between nuclei and mitochondria.

Despite this diversity, conserved trends emerge. All mt-tRNAs have kept features of the primordial RNA adaptors active in ribosome-dependent protein synthesis. This machinery, most of the time specialized in the synthesis of membrane proteins in mitochondria, is confined to peculiar physico-chemical and macromolecular environments. Adaptation to such environments requires *a priori* structural and functional plasticity. This flexibility could rely on adapted recognition rules for the specific interaction of mature tRNAs with their main macromolecular partners, namely aaRSs and mitoribosome. Altogether, this suggests that the structural constraints on mt-encoded tRNAs might be less severe than for cytosolic tRNAs (less modified nts and more conformational flexibility in mt-tRNAs) and that identity rules for aminoacylation are simplified.

Nevertheless, because of its sheer diversity, the number of unanswered questions and of poorly understood processes concerning mt-tRNA biology is huge. A few open questions regarding each of the main chapters of this review are as follows. A first fundamental question relates to the number and nature of tRNAs still encoded in mt-genomes. Why were variable numbers of tRNA genes maintained during evolution? Is this always the result of co-evolution with tRNA import? In some instances imported tRNAs seem to be redundant with mt-encoded tRNAs. Could this mean that these tRNAs might sometimes be involved in processes not related to translation? The tRNA import processes described so far imply that tRNAs are at least partially unfolded during import. Does this mean that these tRNAs are not fully matured and that their structure might be locked inside mitochondria, e.g., through modification events?

Then, another fundamental question is to understand the evolutionary reason for the diversity of mt-tRNA sequences and structures. Why are mt-tRNAs nearly canonical in some phyla while they are peculiar in others? Is this related to phyla-specific diversities in the physiological environment of mitochondria (*i.e.*, local pH, salt, ions and macromolecule concentrations) or is this the result of specific co-evolution processes of tRNAs and tRNA binding proteins in the respective eukaryote groups? Answers might be brought by a more comprehensive characterization of mt-tRNAs across the eukaryote world.

Following this, it will be interesting to determine how the diversity of tRNA maturation systems correlates or not with the diversity of tRNA structures. Have mitochondria evolved independent and specific maturation processes in different eukaryote groups or have ancestral maturation systems rather recruited additional factors to cope with the apparition of non-canonical tRNA structures in some phyla? Another challenge will be to determine to what extent maturation enzymes occur as multifunctional complexes as already observed and how their interactions have implications for the regulation of mt-tRNA biogenesis. Finally, it will be essential to understand if the different mt-tRNA structures are characterized by specific aminoacylation identity rules in the respective eukaryal groups. Answers will be found by a more thorough characterization of mt-tRNAs aminoacylation determinants and antideterminants as well as by aminoacylation kinetics in representative eukaryal phyla.

Answers to questions raised here will help to understand how mitochondrial dysfunctions, often pathogenic in human, can be directly related to mutations in the mt-genome or if they are rather associated with defective tRNA biogenesis machineries. Moreover, the observation that tRNA related dysfunctions are often not directly correlated with strong translational disorders might point out new functions held by tRNAs in mitochondria. Overall, the major challenge of future research on mitochondrial tRNA biology will be to understand if original strategies appeared in mitochondria during eukaryote history as an adaptation to changing mt-genomes and mt-tRNA structures and/or if the tRNAs structures co-evolved with maturation and functional processes to cope with particular mitochondrial environments.

## Supplementary Materials

Supplementary materials can be found at https://www.mdpi.com/1422-0067/16/3/4518/s1.

## References

[1] Russell O., Turnbull D. (2014). Mitochondrial DNA disease-molecular insights and potential routes to a cure. Exp. Cell Res..

[2] Horn R., Gupta K.J., Colombo N. (2014). Mitochondrion role in molecular basis of cytoplasmic male sterility. Mitochondrion.

[3] Schimper A.F.W. (1883). Über die Entwicklung der Chlorophyllkörner und Farbkörper. Bot. Zeitung.

[4] Margulis L. (1975). Symbiotic theory of the origin of eukaryotic organelles; criteria for proof. Symp. Soc. Exp. Biol..

[5] Gray M.W., Burger G., Lang B.F. (1999). Mitochondrial evolution. Science.

[6] Gray M.W. (2014). The pre-endosymbiont hypothesis: A new perspective on the origin and evolution of mitochondria. Cold Spring Harb. Perspect. Biol..

[7] Adams K.L., Palmer J.D. (2003). Evolution of mitochondrial gene content: Gene loss and transfer to the nucleus. Mol. Phylogenet. Evol..

[8] Woodson J.D., Chory J. (2008). Coordination of gene expression between organellar and nuclear genomes. Nat. Rev. Genet..

[9] Dudek J., Rehling P., van der Laan M. (2013). Mitochondrial protein import: Common principles and physiological networks. Biochim. Biophys. Acta.

[10] Salinas T., Duchêne A.-M., Maréchal-Drouard L. (2008). Recent advances in tRNA mitochondrial import. Trends Biochem. Sci..

[11] Alfonzo J.D., Söll D. (2009). Mitochondrial tRNA import—The challenge to understand has just begun. Biol. Chem..

[12] Rubio M.A., Hopper A.K. (2011). Transfer RNA travels from the cytoplasm to organelles. Wiley Interdiscip. Rev. RNA..

[13] Bestwick M.L., Shadel G.S. (2013). Accessorizing the human mitochondrial transcription machinery. Trends Biochem. Sci..

[14] Hammani K., Giegé P. (2014). RNA metabolism in plant mitochondria. Trends Plant Sci..

[15] Huot J.L., Enkler L., Megel C., Karim L., Laporte D., Becker H.D., Duchêne A.-M., Sissler M., Maréchal-Drouard L. (2014). Idiosyncrasies in decoding mitochondrial genomes. Biochimie.

[16] Schneider A., Maréchal-Drouard L. (2000). Mitochondrial tRNA import: Are there distinct mechanisms?. Trends Cell Biol..

[17] Bullerwell C.E., Gray M.W. (2005). *In vitro* characterization of a tRNA editing activity in the mitochondria of *Spizellomyces punctatus*, a *Chytridiomycete* fungus. J. Biol. Chem..

[18] Salinas T., El Farouk-Ameqrane S., Ubrig E., Sauter C., Duchêne A.-M., Maréchal-Drouard L. (2014). Molecular basis for the differential interaction of plant mitochondrial VDAC proteins with tRNAs. Nucleic Acids Res..

[19] Sloan D.B., Alverson A.J., Chuckalovcak J.P., Wu M., McCauley D.E., Palmer J.D., Taylor D.R. (2012). Rapid evolution of enormous, multichromosomal genomes in flowering plant mitochondria with exceptionally high mutation rates. PLoS Biol..

[20] Fey J., Dietrich A., Cosset A., Desprez T., Maréchal-Drouard L. (1997). Evolutionary aspects of “Chloroplast-like” trnN and trnH expression in higher-plant mitochondria. Curr. Genet..

[21] Kitazaki K., Kubo T., Kagami H., Matsumoto T., Fujita A., Matsuhira H., Matsunaga M., Mikami T. (2011). A horizontally transferred tRNA^Cys^ gene in the sugar beet mitochondrial genome: Evidence that the gene is present in diverse angiosperms and its transcript is aminoacylated. Plant J..

[22] Schneider A. (2011). Mitochondrial tRNA import and its consequences for mitochondrial translation. Annu. Rev. Biochem..

[23] Rubio M.A., Rinehart J.J., Krett B., Duvezin-Caubet S., Reichert A.S., Söll D., Alfonzo J.D. (2008). Mammalian mitochondria have the innate ability to import tRNAs by a mechanism distinct from protein import. Proc. Nalt. Acad. Sci. USA.

[24] Dörner M., Altmann M., Pääbo S., Mörl M. (2001). Evidence for import of a lysyl-tRNA into marsupial mitochondria. Mol. Biol. Cell.

[25] Martin R.P., Schneller J.-M., Stahl A.J., Dirheimer G. (1979). Import of nuclear deoxyribonucleic acid coded lysine-accepting transfer ribonucleic acid (anticodon C-U-U) into yeast mitochondria. Biochemistry.

[26] Rinehart J., Krett B., Rubio M.-A.T., Alfonzo J.D., Söll D. (2005). *Saccharomyces cerevisiae* imports the cytosolic pathway for gln-tRNA synthesis into the mitochondion. Genes Dev..

[27] Oda K., Yamato K., Ohta E., Nakamura Y., Takemura M., Nozato N., Akashi K., Ohyama K. (1992). Transfer RNA genes in the mitochondrial genome from a liverwort, *Marchantia polymorpha*: The absence of chloroplast-like tRNAs. Nucleic Acids Res..

[28] Akashi K., Sakurai K., Hirayama J., Fukuzawa H., Ohyama K. (1996). Occurrence of nuclear-encoded tRNA^Ile^ in mitochondria of the liverwort *Marchantia polymorpha*. Curr. Genet..

[29] Akashi K., Hirayama J., Takenaka M., Yamaoka S., Suyama Y., Fukuzawa H., Ohyama K. (1997). Accumulation of nuclear-encoded tRNA^Thr(AGU)^ in mitochondria of the liverwort *Marchantia polymorpha*. Biochim. Biophys. Acta.

[30] Akashi K., Takenaka M., Yamaoka S., Suyama Y., Fukuzawa H., Ohyama K. (1998). Coexistence of nuclear DNA-encoded tRNA^Val(AAC)^ and mitochondrial DNA-encoded tRNA^Val(UAC)^ in mitochondria of a liverwort *Marchantia polymorpha*. Nucleic Acids Res..

[31] Chen H.C., Viry-Moussaid M., Dietrich A., Wintz H. (1997). Evolution of a mitochondrial tRNA^Phe^ gene in *A. thaliana*: Import of cytosolic tRNA phe into mitochondria. Biochem. Biophys. Res. Commun..

[32] Duchêne A.-M., Maréchal-Drouard L. (2001). The chloroplast-derived trnw and trnM-e genes are not expressed in *Arabidopsis* mitochondria. Biochem. Biophys. Res. Commun..

[33] Dietrich A., Maréchal-Drouard L., Carneiro V., Cosset A., Small I. (1996). A single base change prevents import of cytosolic tRNA^Ala^ into mitochondria in transgenic plants. Plant J..

[34] Delage L., Dietrich A., Cosset A., Maréchal-Drouard L. (2003). *In vitro* import of a nuclearly encoded tRNA into mitochondria of *Solanum tuberosum*. Mol. Cell. Biol..

[35] Laforest M.J., Delage L., Maréchal-Drouard L. (2005). The T-domain of cytosolic tRNA^Val^, an essential determinant for mitochondrial import. FEBS Lett..

[36] Salinas T., Schaeffer C., Maréchal-Drouard L., Duchêne A.-M. (2005). Sequence dependence of tRNA^Gly^ import into tobacco mitochondria. Biochimie.

[37] Maréchal-Drouard L., Guillemaut P., Cosset A., Arbogast M., Weber F., Weil J.-H., Dietrich A. (1990). Transfer RNAs of potato (*Solanum tuberosum*) mitochondria have different genetic origins. Nucleic Acids Res..

[38] Small I., Maréchal-Drouard L., Masson J., Pelletier G., Cosset A., Weil J.-H., Dietrich A. (1992). *In vivo* import of a normal or mutagenized heterologous transfer RNA into mitochondria of transgenic plants: Towards novel ways of influencing mitochondrial gene expression?. EMBO J..

[39] Kumar R., Maréchal-Drouard L., Akama K., Small I. (1996). Striking differences in mitochondrial tRNA import between different plant species. Mol. Gen. Genet..

[40] Brubacher-Kauffmann S., Maréchal-Drouard L., Cosset A., Dietrich A., Duchêne A.-M. (1999). Differential import of nuclear-encoded tRNA^Gly^ isoacceptors into *Solanum tuberosum* mitochondria. Nucleic Acids Res..

[41] Glover K.E., Spencer D.F., Gray M.W. (2001). Identification and structural characterization of nucleus-encoded transfer RNAs imported into wheat mitochondria. J. Biol. Chem..

[42] Vinogradova E., Salinas T., Cognat V., Remacle C., Maréchal-Drouard L. (2009). Steady-state levels of imported tRNAs in *Chlamydomonas* mitochondria are correlated with both cytosolic and mitochondrial codon usages. Nucleic Acids Res..

[43] Suyama Y. (1967). The origins of mitochondrial ribonucleic acids in *Tetrahymena pyriformis*. Biochemistry.

[44] Chiu N., Chiu A., Suyama Y. (1975). Native and imported transfer RNA in mitochondria. J. Mol. Biol..

[45] Suyama Y. (1986). Two dimensional polyacrylamide gel electrophoresis analysis of *Tetrahymena* mitochondrial tRNA. Curr. Genet..

[46] Simpson A.M., Suyama Y., Dewes H., Campbell D.A., Simpson L. (1989). Kinetoplastid mitochondria contain functional tRNAs which are encoded in nuclear DNA and also contain small minicircle and maxicircle transcripts of unknown function. Nucleic Acids Res..

[47] Lye L.F., Chen D.H., Suyama Y. (1993). Selective import of nuclear-encoded tRNAs into mitochondria of the protozoan *Leishmania tarentolae*. Mol. Biochem. Parasitol..

[48] Shi X., Chen D.H., Suyama Y. (1994). A nuclear tRNA gene cluster in the protozoan *Leishmania tarentolae* and differential distribution of nuclear-encoded tRNAs between the cytosol and mitochondria. Mol. Biochem. Parasitol..

[49] Hancock K., Hajduk S.L. (1990). The mitochondrial tRNAs of *Trypanosoma brucei* are nuclear encoded. J. Biol. Chem..

[50] Schneider A., McNally K.P., Agabian N. (1994). Nuclear-encoded mitochondrial tRNAs of *Trypanosoma brucei* have a modified cytidine in the anticodon loop. Nucleic Acids Res..

[51] Sharma A., Sharma A. (2015). *Plasmodium falciparum* mitochondria import tRNAs along with an active phenylalanyl-tRNA synthetase. Biochem. J..

[52] Entelis N., Kolesnikova O., Kazakova H., Brandina I., Kamenski P., Martin R.P., Tarassov I. (2002). Import of nuclear encoded rnas into yeast and human mitochondria: Experimental approaches and possible biomedical applications. Genet. Eng..

[53] Duchêne A.-M., Pujol C., Maréchal-Drouard L. (2009). Import of tRNAs and aminoacyl-tRNA synthetases into mitochondria. Curr. Genet..

[54] Lithgow T., Schneider A. (2010). Evolution of macromolecular import pathways in mitochondria, hydrogenosomes and mitosomes. Philos. Trans. R. Soc. Lond. B Biol. Sci..

[55] Sieber F., Duchêne A.-M., Maréchal-Drouard L. (2011). Mitochondrial rna import: From diversity of natural mechanisms to potential applications. Int. Rev. Cell Mol. Biol..

[56] Kamenski P., Kolesnikova O., Jubenot V., Entelis N., Krasheninnikov I.A., Martin R.P., Tarassov I. (2007). Evidence for an adaptation mechanism of mitochondrial translation via tRNA import from the cytosol. Mol. Cell.

[57] Entelis N., Brandina I., Kamenski P., Krasheninnikov I.A., Martin R.P., Tarassov I. (2006). A glycolytic enzyme, enolase, is recruited as a cofactor of tRNA targeting toward mitochondria in *Saccharomyces cerevisiae*. Genes Dev..

[58] Kamenski P., Smirnova E., Kolesnikova O., Krasheninnikov I.A., Martin R.P., Entelis N., Tarassov I. (2010). tRNA mitochondrial import in yeast: Mapping of the import determinants in the carrier protein, the precursor of mitochondrial lysyl-tRNA synthetase. Mitochondrion.

[59] Rusconi C.P., Cech T.R. (1996). The anticodon is the signal sequence for mitochondrial import of glutamine tRNA in *Tetrahymena*. Genes Dev..

[60] Crausaz Esseiva A., Maréchal-Drouard L., Cosset A., Schneider A. (2004). The T-stem determines the cytosolic or mitochondrial localization of trypanosomal tRNAs^Met^. Mol. Biol. Cell.

[61] Bouzaidi-Tiali N., Aeby E., Charriere F., Pusnik M., Schneider A. (2007). Elongation factor 1a mediates the specificity of mitochondrial tRNA import in *T. brucei*. EMBO J..

[62] Delage L., Duchêne A.-M., Zaepfel M., Maréchal-Drouard L. (2003). The anticodon and the D-domain sequences are essential determinants for plant cytosolic tRNA^Val^ import into mitochondria. Plant J..

[63] Salinas K., Wierzbicki S., Zhou L., Schmitt M.E. (2005). Characterization and purification of *Saccharomyces cerevisiae* RNase MRP reveals a new unique protein component. J. Biol. Chem..

[64] Kapushoc S.T., Alfonzo J.D., Simpson L. (2002). Differential localization of nuclear-encoded tRNAs between the cytosol and mitochondrion in *Leishmania tarentolae*. RNA.

[65] Suyama Y., Wong S., Campbell D.A. (1998). Regulated tRNA import in *Leishmania* mitochondria. Biochim. Biophys. Acta.

[66] Tan T.H., Pach R., Crausaz A., Ivens A., Schneider A. (2002). tRNAs in *Trypanosoma brucei*: Genomic organization, expression, and mitochondrial import. Mol. Cell. Biol..

[67] Entelis N.S., Kolesnikova O.A., Dogan S., Martin R.P., Tarassov I.A. (2001). 5S rRNA and tRNA import into human mitochondria: Comparison of *in vitro* requirements. J. Biol. Chem..

[68] Salinas T., Duby F., Larosa V., Coosemans N., Bonnefoy N., Motte P., Maréchal-Drouard L., Remacle C. (2012). Co-evolution of mitochondrial tRNA import and codon usage determines translational efficiency in the green alga *Chlamydomonas*. PLoS Genet..

[69] Tarassov I., Entelis N., Martin R.P. (1995). An intact protein translocating machinery is required for mitochondrial import of a yeast cytoplasmic tRNA. J. Mol. Biol..

[70] Kolesnikova O., Kazakova H., Comte C., Steinberg S., Kamenski P., Martin R.P., Tarassov I., Entelis N. (2010). Selection of RNA aptamers imported into yeast and human mitochondria. RNA.

[71] Brandina I., Smirnov A., Kolesnikova O., Entelis N., Krasheninnikov I.A., Martin R.P., Tarassov I. (2007). tRNA import into yeast mitochondria is regulated by the ubiquitin-proteasome system. FEBS Lett..

[72] Salinas T., Duchêne A.-M., Delage L., Nilsson S., Glaser E., Zaepfel M., Maréchal-Drouard L. (2006). The voltage-dependent anion channel, a major component of the tRNA import machinery in plant mitochondria. Proc. Natl. Acad. Sci. USA.

[73] Dietrich A., Small I., Cosset A., Weil J.-H., Maréchal-Drouard L. (1996). Editing and import: Strategies for providing plant mitochondria with a complete set of functional transfer RNAs. Biochimie.

[74] Sieber F., Placido A., El Farouk-Ameqrane S., Duchêne A.-M., Maréchal-Drouard L. (2011). A protein shuttle system to target RNA into mitochondria. Nucleic Acids Res..

[75] Schekman R. (2010). Editorial expression of concern and correction. Proc. Nalt. Acad. Sci. USA.

[76] Pusnik M., Charriere F., Maser P., Waller R.F., Dagley M.J., Lithgow T., Schneider A. (2009). The single mitochondrial porin of *Trypanosoma brucei* is the main metabolite transporter in the outer mitochondrial membrane. Mol. Biol. Evol..

[77] Seidman D., Johnson D., Gerbasi V., Golden D., Orlando R., Hajduk S. (2012). Mitochondrial membrane complex that contains proteins necessary for tRNA import in *Trypanosoma brucei*. J. Biol. Chem..

[78] Tschopp F., Charriere F., Schneider A. (2011). *In vivo* study in *trypanosoma brucei* links mitochondrial transfer rna import to mitochondrial protein import. EMBO Rep..

[79] Heckman J.E., Hecker L.I., Schwartzbach S.D., Barnett W.E., Baumstark B., RajBhandary U.L. (1978). Structure and function of initiator methionine tRNA from the mitochondria of *Neurospora crassa*. Cell.

[80] Heckman J.E., Alzner-Deweerd B., RajBhandary U.L. (1979). Interesting and unusual features in the sequence of *Neurospora crassa* mitochondrial tyrosine transfer RNA. Proc. Natl. Acad. Sci. USA.

[81] Sibler A.P., Martin R.P., Dirheimer G. (1979). The nucleotide sequence of yeast mitochondrial histidine-tRNA. FEBS Lett..

[82] Martin R.P., Sibler A.P., Schneller J.-M., Keith G., Stahl A.J., Dirheimer G. (1978). Primary structure of yeast mitochondrial DNA-coded phenylalanine-tRNA. Nucleic Acids Res..

[83] Maréchal L., Guillemaut P., Grienenberger J.-M., Jeannin G., Weil J.-H. (1985). Structure of bean mitochondrial transfer RNA^Phe^ and localization of the transfer RNA^Phe^ gene on the mitochondrial genomes of maize and wheat. FEBS Lett..

[84] Maréchal L., Guillemaut P., Grienenberger J.-M., Jeannin G., Weil J.-H. (1985). Sequence and codon recognition of bean mitochondria and chloroplast tRNAs^Trp^: Evidence for a high degree of homology. Nucleic Acid Res..

[85] Maréchal L., Guillemaut P., Weil J.-H. (1985). Sequences of two bean mitochondria tRNAs^Tyr^ which differ in the level of post-transcriptional modification and have a prokaryotic-like large extra-loop. Plant Mol. Biol..

[86] Dirheimer G., Keith G., Sibler A.-P., Martin R.P., Schimmel P., Söll D., Abelson J. (1979). The primary structure of tRNAs and their rare nucleosides. Transfer RNA: Structure, Properties and Recognition.

[87] RajBhandary U.L., Heckman J.E., Yin S., Alzner-DeWeerd B., Ackerman E., Schimmel P., Söll D., Abelson J. (1979). Recent develpments in tRNA sequencing methods as applied to analyses of mitochondrial tRNAs. Transfer RNA: Structure, Properties and Recognition.

[88] Arcari P., Brownlee G.G. (1980). The nucleotide sequence of a small (3S) seryl-tRNA (anticodon GCU) from beef heart mitochondria. Nucleic Acids Res..

[89] De Bruijn M.H., Schreier P.H., Eperon I.C., Barrell B.G., Chen E.Y., Armstrong P.W., Wong J.F., Roe B.A. (1980). A mammalian mitochondrial serine transfer RNA lacking the “Dihydrouridine” loop and stem. Nucleic Acids Res..

[90] Pütz J., Dupuis B., Sissler M., Florentz C. (2007). Mamit-tRNA, a database of mammalian mitochondrial tRNA primary and secondary structures. RNA.

[91] Cantara W.A., Crain P.F., Rozenski J., McCloskey J.A., Harris K.A., Zhang X., Vendeix F.A., Fabris D., Agris P.F. (2011). The RNA modification database, RNAmdb: 2011 update. Nucleic Acids Res..

[92] Giegé R., Jühling F., Pütz J., Stadler P., Sauter C., Florentz C. (2012). Structure of transfer RNAs: Similarity and variability. Wiley Interdiscip. Rev. RNA.

[93] Wolstenholme D.R., Macfarlane J.L., Okimoto R., Clary D.O., Wahleithner J.A. (1987). Bizarre tRNAs inferred from DNA sequences of mitochondrial genomes of nematode worms. Proc. Natl. Acad. Sci. USA.

[94] Jühling F., Pütz J., Florentz C., Stadler P.F. (2012). Armless mitochondrial tRNAs in *Enoplea* (*Nematoda*). RNA Biol..

[95] Palopoli M.F., Minot S., Pei D., Satterly A., Endrizzi J. (2014). Complete mitochondrial genomes of the human follicle mites *Demodex brevis* and *D. folliculorum*: Novel gene arrangement, truncated tRNA genes, and ancient divergence between species. BMC Genomics.

[96] Wende S., Platzer E.G., Juhling F., Pütz J., Florentz C., Stadler P.F., Mörl M. (2013). Biological evidence for the world’s smallest tRNAs. Biochimie.

[97] Chen D.-S., Jin P.Y., Zhang K.J., Ding X.L., Yang S.X., Ju J.F., Zhao J.Y., Hong X.Y. (2014). The complete mitochondrial genomes of six species of *Tetranychus* provide insights into the phylogeny and evolution of spider mites. PLoS One.

[98] Watanabe K. (2010). Unique features of animal mitochondrial translation systems—The non-universal genetic code, unusual features of the translational apparatus and their relevance to human mitochondrial diseases. Proc. Jpn. Acad. Series B-Phys. Biol. Sci..

[99] Grosjean H. (2009). DNA and RNA Modification Enzymes: Structure, Mechanisms, Function, and Evolution.

[100] Machnicka M.A., Milanowska K., Osman Oglou O., Purta E., Kurkowska M., Olchowik A., Januszewski W., Kalinowski S., Dunin-Horkawicz S., Rother K.M. (2013). Modomics: A database of rna modification pathways–2013 update. Nucleic Acids Res..

[101] Suzuki T., Suzuki T. (2014). A complete landscape of post-transcriptional modifications in mammalian mitochondrial tRNAs. Nucleic Acids Res..

[102] Jackman J.E., Alfonzo J.D. (2013). Transfer RNA modifications: Nature’s combinatorial chemistry playground. Wiley Interdiscip. Rev. RNA.

[103] Moriya J., Yokogawa T., Wakita K., Ueda T., Nishikawa K., Crain P.F., Hashizume T., Pomerantz S.C., MacCloskey J.A., Kawai G. (1994). A novel modified nucleoside found at the first position of the anticodon of methionine tRNA from bovine liver mitochondria. Biochemistry.

[104] Watanabe Y., Tsurui H., Ueda T., Furushima R., Takamiya S., Kita K., Nishikawa K., Watanabe K. (1994). Primary and higher order structures of nematode (*Ascaris suum*) mitochondrial tRNAs lacking either the T or D stem. J. Biol. Chem..

[105] Suzuki T., Suzuki T., Wada T., Saigo K., Watanabe K. (2002). Taurine as a constituent of mitochondrial tRNAs: New insights into the functions of taurine and human mitochondrial diseases. EMBO J..

[106] Weber F., Dietrich A., Weil J.-H., Maréchal-Drouard L. (1990). A potato mitochondrial isoleucine tRNA is coded by a mitochondrial gene possessing a methionine anticodon. Nucleic Acid Res..

[107] Dubin D.T., HsuChen C.-C., Cleaves G.R., Timko K.D. (1984). Sequence and structure of a serine transfer RNA with GCU anticodon from mosquito mitochondria. J. Mol. Biol..

[108] Suzuki T., Miyauchi K., Yokobori S.I., Shigi N., Kondow A., Takeuchi N., Yamagishi A., Watanabe K. (2011). Taurine-containing uridine modifications in tRNA anticodons are required to decipher non-universal genetic codes in ascidian mitochondria. J. Biol. Chem..

[109] Helm M., Brulé H., Degoul F., Cepanec C., Leroux J.-P., Giegé R., Florentz C. (1998). The presence of modified nucleotides is required for cloverleaf folding of a human mitochondrial tRNA. Nucleic Acids Res..

[110] Voigts-Hoffmann F., Hengesbach M., Kobitski A.Y., van Aerschot A., Herdewijn P., Nienhaus G.U., Helm M. (2007). A methyl group controls conformational equilibrium in human mitochondrial tRNA^Lys^. J. Am. Chem. Soc..

[111] Hayashi I., Kawai G., Watanabe K. (1998). Higher-order structure and thermal instability of bovine mitochondrial tRNA^Ser(UGA)^ investigated by proton NMR spectroscopy. J. Mol. Biol..

[112] Steinberg S., Cedergren R. (1994). Structural compensation in atypical mitochondrial tRNAs. Nat. Struct. Biol..

[113] Steinberg S., Leclerc F., Cedergren R. (1997). Structural rules and conformational compensations in the tRNA L-form. J. Mol. Biol..

[114] Lang B.F., Lavrov D., Beck N., Steinberg S.V., Bullerwell C.E. (2012). Mitochondrial tRNA structure, identity, and evolution of the genetic code. Organelle Genetics. Evolution of Organelle Genomes and Gene Expression.

[115] Leehey M.A., Squassoni C.A., Friederich M.W., Mills J.B., Hagerman P.J. (1995). A noncanonical tertiary conformation of a human mitochondrial transfer RNA. Biochemistry.

[116] Frazer-Abel A.A., Hagerman P.J. (1999). Determination of the angle between the acceptor and anticodon stems of a truncated mitochondrial tRNA. J. Mol. Biol..

[117] Nozawa K., O'Donoghue P., Gundllapalli S., Araiso Y., Ishitani R., Umehara T., Söll D., Nureki O. (2009). Pyrrolysyl-tRNA synthetase-tRNA^Pyl^ structure reveals the molecular basis of orthogonality. Nature.

[118] Théobald-Dietrich A., Frugier M., Giegé R., Rudinger-Thirion J. (2004). Atypical archaeal tRNA pyrrolysine transcript behaves towards EF-Tu as a typical elongator tRNA. Nucleic Acids Res..

[119] Ohtsuki T., Kawai G., Watanabe K. (2002). The minimal tRNA: Unique structure of *Ascaris suum* mitochondrial tRNA^Ser(UCU)^ having a short T arm and lacking the entire D arm. FEBS Lett..

[120] Messmer M., Pütz J., Suzuki T., Suzuki T., Sauter C., Sissler M., Florentz C. (2009). Tertiary network in mammalian mitochondrial tRNA^Asp^ revealed by solution probing and phylogeny. Nucleic Acids Res..

[121] Wolfson A.D., Khvorova A.M., Sauter C., Florentz C., Giegé R. (1999). Mimics of yeast tRNA^Asp^ and their recognition by aspartyl-tRNA synthetase. Biochemistry.

[122] Lusic H., Gustilo E.M., Vendeix F.A., Kaiser R., Delaney M.O., Graham W.D., Moye V.A., Cantara W.A., Agris P.F., Deiters A. (2008). Synthesis and investigation of the 5-formylcytidine modified, anticodon stem and loop of the human mitochondrial tRNA^Met^. Nucleic Acids Res..

[123] Bilbille Y., Gustilo E.M., Harris K.A., Jones C.N., Lusic H., Kaiser R.J., Delaney M.O., Spremulli L.L., Deiters A., Agris P.F. (2011). The human mitochondrial tRNA^Met^: Structure/function relationship of a unique modification in the decoding of unconventional codons. J. Mol. Biol..

[124] Cantara W.A., Murphy F.V., Demirci H., Agris P.F. (2013). Expanded use of sense codons is regulated by modified cytidines in tRNA. Proc. Natl. Acad. Sci. USA.

[125] Jühling F., Mörl M., Hartmann R., Sprinzl M., Stadler P.F., Pütz J. (2009). Compilation of tRNA sequences and tRNA genes. Nucleic Acids Res..

[126] Schnare M.N., Heinonen T.Y., Young P.G., Gray M.W. (1985). Phenylalanine and tyrosine transfer RNAs encoded by *Tetrahymena pyriformis* mitochondrial DNA: Primary sequence, post-transcriptional modifications, and gene localization. Curr. Genet..

[127] Cognat V., Pawlak G., Duchêne A.-M., Daujat M., Gigant A., Salinas T., Michaud M., Gutmann B., Giegé P., Gobert A. (2013). PlantRNA, a database for tRNAs of photosynthetic eukaryotes. Nucleic Acids Res..

[128] Schnare M.N., Greenwood S.J., Gray M.W. (1995). Primary sequence and post-transcriptional modification pattern of an unusual mitochondrial tRNA^Met^ from Tetrahymena pyriformis. FEBS Lett..

[129] Masta S.E., Boore J.L. (2008). Parallel evolution of truncated transfer RNA genes in arachnid mitochondrial genomes. Mol. Biol. Evol..

[130] Masta S.E., McCall A., Longhorm S.J. (2010). Rare genomic changes and mitochondrial sequences provide independent support for congruent relationships among the sea spiders (*Arthropoda*, *Pycnogonida*).

[131] Auffinger P., Westhof E. (2001). An extended structural signature for the tRNA anticodon loop. RNA.

[132] Durant P.C., Davis D.R. (1999). Stabilization of the anticodon stem-loop of tRNA^Lys, 3^ by an A+-C base-pair and by pseudouridine. J. Mol. Biol..

[133] Klipcan L., Moor N., Finarov I., Kessler N., Sukhanova M., Safro M.G. (2012). Crystal structure of human mitochondrial phers complexed with tRNA^Phe^ in the active “Open” state. J. Mol. Biol..

[134] Chimnaronk S., Gravers Jeppesen M., Suzuki T., Nyborg J., Watanabe K. (2005). Dual-mode recognition of noncanonical tRNAs^Ser^ by seryl-tRNA synthetase in mammalian mitochondria. EMBO J..

[135] Neuenfeldt A., Lorber B., Ennifar E., Gaudry A., Sauter C., Sissler M., Florentz C. (2013). Thermodynamic properties distinguish human mitochondrial aspartyl-tRNA synthetase from bacterial homolog with same 3D architecture. Nucleic Acids Res..

[136] Ling J., Roy H., Qin D., Rubio M.A., Alfonzo J.D., Fredrick K., Ibba M. (2007). Pathogenic mechanism of a human mitochondrial tRNA^Phe^ mutation associated with myoclonic epilepsy with ragged red fibers syndrome. Proc. Natl. Acad. Sci. USA.

[137] Goldgur Y., Mosyak L., Reshetnikova L., Ankilova V., Lavrik O., Khodyreva S., Safro M. (1997). The crystal structure of phenylalanyl-tRNA synthetase from *Thermus thermophilus* complexed with cognate tRNA^Phe^. Structure.

[138] Gobert A., Pinker F., Fuchsbauer O., Gutmann B., Boutin R., Roblin P., Sauter C., Giegé P. (2013). Structural insights into protein-only RNase P complexed with tRNA. Nat. Commun..

[139] Agrawal R.K., Sharma M.R. (2012). Structural aspects of mitochondrial translational apparatus. Curr. Opin. Struct. Biol..

[140] Greber B.J., Boehringer D., Leitner A., Bieri P., Voigts-Hoffmann F., Erzberger J.P., Leibundsgut M., Aebersold M., Ban N. (2014). Architecture of the large subunit of the mammalian mitochondrial ribosome. Nature.

[141] Amunts A., Brown A., Bai X.C., Llacer J.L., Hussain T., Emsley P., Long F., Murshudov G., Scheres S.H., Ramakrishnan V. (2014). Structure of the yeast mitochondrial large ribosomal subunit. Science.

[142] Anderson S., Bankier A.T., Barrel B.G., de Bruijn M.H.L., Coulson A.R., Drouin J., Eperon J.C., Nierlich D.P., Roe B.A., Sanger F. (1981). Sequence and organization of the human mitochondrial genome. Nature.

[143] Ojala D., Montoya J., Attardi G. (1981). tRNA punctuation model of RNA processing in human mitochondria. Nature.

[144] Rossmanith W. (2012). Of P and Z: Mitochondrial tRNA processing enzymes. Biochim. Biophys. Acta.

[145] Unseld M., Marienfeld J.R., Brandt P., Brennicke A. (1997). The mitochondrial genome of *Arabidopsis thaliana* contains 57 genes in 366,924 nucleotides. Nat. Genet..

[146] Ringel R., Sologub M., Morozov Y.I., Litonin D., Cramer P., Temiakov D. (2011). Structure of human mitochondrial RNA polymerase. Nature.

[147] Schwinghammer K., Cheung A.C., Morozov Y.I., Agaronyan K., Temiakov D., Cramer P. (2013). Structure of human mitochondrial RNA polymerase elongation complex. Nat. Struct. Mol. Biol..

[148] Giegé P. (2013). Pentatricopeptide repeat proteins: A set of modular RNA-specific binders massively used for organelle gene expression. RNA Biol..

[149] Kühn K., Richter U., Meyer E.H., Delannoy E., de Longevialle A.F., O'Toole N., Borner T., Millar A.H., Small I.D., Whelan J. (2009). Phage-type RNA polymerase rpotmp performs gene-specific transcription in mitochondria of *Arabidopsis thaliana*. Plant Cell.

[150] Gutmann B., Gobert A., Giegé P., Drouard L. (2012). Mitochondrial genome evolution and the emergence of PPR proteins. Mitochondrial Genome Evolution.

[151] Lang B.F., Burger G., O’Kelly C.J., Cedergren R., Golding G.B., Lemieux C., Sankoff D., Turmel M., Gray M.W. (1997). An ancestral mitochondrial DNA resembling a eubacterial genome in miniature. Nature.

[152] Altman S. (2007). A view of RNase P. Mol. Biosyst..

[153] Pinker F., Bonnard G., Gobert A., Gutmann B., Hammani K., Sauter C., Gegenheimer P.A., Giegé P. (2013). PPR proteins shed a new light on RNase P biology. RNA Biol..

[154] Seif E.R., Forget L., Martin N.C., Lang B.F. (2003). Mitochondrial RNase P RNAs in ascomycete fungi: Lineage-specific variations in RNA secondary structure. RNA.

[155] Li D., Willkomm D.K., Schön A., Hartmann R.K. (2007). RNase P of the *Cyanophora paradoxa* cyanelle: A plastid ribozyme. Biochimie.

[156] Burger G., Gray M.W., Forget L., Lang B.F. (2013). Strikingly bacteria-like and gene-rich mitochondrial genomes throughout jakobid protists. Genome Biol. Evol..

[157] Hartmann E., Hartmann R.K. (2003). The enigma of ribonuclease P evolution. Trends Genet..

[158] Holzmann J., Frank P., Loffler E., Bennett K.L., Gerner C., Rossmanith W. (2008). RNase P without RNA: Identification and functional reconstitution of the human mitochondrial tRNA processing enzyme. Cell.

[159] Gobert A., Gutmann B., Taschner A., Gossringer M., Holzmann J., Hartmann R.K., Rossmanith W., Giegé P. (2010). A single *Arabidopsis* organellar protein has RNase P activity. Nat. Struct. Mol. Biol..

[160] Taschner A., Weber C., Buzet A., Hartmann R.K., Hartig A., Rossmanith W. (2012). Nuclear RNase P of *Trypanosoma brucei*: A single protein in place of the multicomponent RNA-protein complex. Cell Rep..

[161] Sugita C., Komura Y., Tanaka K., Kometani K., Satoh H., Sugita M. (2014). Molecular characterization of three PRORP proteins in the moss *Physcomitrella patens*: Nuclear PRORP protein is not essential for moss viability. PLoS One.

[162] Gutmann B., Gobert A., Giegé P. (2012). PRORP proteins support RNase P activity in both organelles and the nucleus in *Arabidopsis*. Genes Dev..

[163] Pavlova L.V., Gossringer M., Weber C., Buzet A., Rossmanith W., Hartmann R.K. (2012). tRNA processing by protein-only *versus* RNA-based RNase P: Kinetic analysis reveals mechanistic differences. Chembiochem..

[164] Vogel A., Schilling O., Spath B., Marchfelder A. (2005). The tRNase Z family of proteins: Physiological functions, substrate specificity and structural properties. Biol. Chem..

[165] Rossmanith W. (2011). Localization of human RNase Z isoforms: Dual nuclear/mitochondrial targeting of the elac2 gene product by alternative translation initiation. PLoS One.

[166] Duchêne A.-M., Giegé P. (2012). Dual localized mitochondrial and nuclear proteins as gene expression regulators in plants. Front. Plant Sci..

[167] Canino G., Bocian E., Barbezier N., Echeverria M., Forner J., Binder S., Marchfelder A. (2009). *Arabidopsis* encodes four tRNase Z enzymes. Plant Physiol..

[168] Vörtler S., Mörl M. (2010). tRNA-nucleotidyltransferases: Highly unusual RNA polymerases with vital functions. FEBS Lett..

[169] Reichert A.S., Thurlow D.L., Mörl M. (2001). A eubacterial origin for the human tRNA nucleotidyltransferase?. Biol. Chem..

[170] Hammani K., Bonnard G., Bouchoucha A., Gobert A., Pinker F., Salinas T., Giegé P. (2014). Helical repeats modular proteins are major players for organelle gene expression. Biochimie.

[171] Benne R., Van den Burg J., Brakenhoff J.P., Sloof P., Van Boom J.H., Tromp M.C. (1986). Major transcript of the frameshifted coxii gene from trypanosome mitochondria contains four nucleotides that are not encoded in the DNA. Cell.

[172] Knoop V. (2011). When you can’t trust the DNA: RNA editing changes transcript sequences. Cell. Mol. Life Sci..

[173] Phizicky E.M., Alfonzo J.D. (2010). Do all modifications benefit all tRNAs?. FEBS Lett..

[174] Torres A.G., Batlle E., Ribas de Pouplana L. (2014). Role of tRNA modifications in human diseases. Trends Mol. Med..

[175] Yasukawa T., Suzuki T., Ishii N., Ueda T., Ohta S., Watanabe K. (2000). Defect in modification at the anticodon wobble nucleotide of mitochondrial tRNA^Lys^ with the MERRF encephalomyopathy pathogenic mutation. FEBS Lett..

[176] Kirino Y., Yasukawa T., Marjavaara S.K., Jacobs H.T., Holt I.J., Watanabe K., Suzuki T. (2006). Acquisition of the wobble modification in mitochondrial tRNA^Leu(CUN)^ bearing the G12300A mutation suppresses the MELAS molecular defect. Hum. Mol. Gen..

[177] Suzuki T., Nagao A. (2011). Human mitochondrial diseases caused by lack of taurine modification in mitochondrial tRNAs. Wiley Interdiscip. Rev. RNA.

[178] Umeda N., Suzuki T., Yukawa M., Ohya Y., Shindo H., Watanabe K. (2005). Mitochondria-specific rna-modifying enzymes responsible for the biosynthesis of the wobble base in mitochondrial tRNAs. Implications for the molecular pathogenesis of human mitochondrial diseases. J. Biol. Chem..

[179] Vilardo E., Nachbagauer C., Buzet A., Taschner A., Holzmann J., Rossmanith W. (2012). A subcomplex of human mitochondrial RNase P is a bifunctional methyltransferase--extensive moonlighting in mitochondrial tRNA biogenesis. Nucleic Acids Res..

[180] Yokobori S.I., Pääbo S. (1995). tRNA editing in metazoans. Nature.

[181] Börner G.V., Yokobori S.-I., Mörl M., Dörner M., Pääbo S. (1997). RNA editing in metazoan mitochondria: Staying fit without sex. FEBS Lett..

[182] Reichert A., Rothbauer U., Mörl M. (1998). Processing and editing of overlapping tRNAs in human mitochondria. J. Biol. Chem..

[183] Lonergan K.M., Gray M.W. (1993). Editing of transfer rnas in *Acanthamoeba castellanii* mitochondria. Science.

[184] Laforest M.J., Bullerwell C.E., Forget L., Lang B.F. (2004). Origin, evolution, and mechanism of 5' tRNA editing in chytridiomycete fungi. RNA.

[185] Antes T., Costandy H., Mahendran R., Spottswood M., Miller D. (1998). Insertional editing of mitochondrial tRNAs of *Physarum polycephalum* and *Didymium nigripes*. Mol. Cell. Biol..

[186] Börner G.V., Mörl M., Janke A., Pääbo S. (1996). RNA editing changes the identity of a mitochondrial tRNA in marsupials. EMBO J..

[187] Giegé P., Brennicke A. (1999). RNA editing in *Arabidopsis* mitochondria effects 441 C to U changes in ORFs. Proc. Natl. Acad. Sci. USA.

[188] Takenaka M., Zehrmann A., Verbitskiy D., Hartel B., Brennicke A. (2013). RNA editing in plants and its evolution. Annu. Rev. Genet..

[189] Fey J., Weil J.-H., Tomita K., Cosset A., Dietrich A., Small I., Maréchal-Drouard L. (2001). Editing of plant mitochondrial transfer RNAs. Acta Biochim. Pol..

[190] Maréchal-Drouard L., Cosset A., Remacle C., Ramamonjisoa D., Dietrich A. (1996). A single editing event is a prerequisite for efficient processing of potato mitochondrial phenylalanine tRNA. Mol. Cell. Biol..

[191] Barkan A., Small I. (2014). Pentatricopeptide repeat proteins in plants. Annu. Rev. Plant Biol..

[192] Alfonzo J.D., Blanc V., Estevez A.M., Rubio M.A., Simpson L. (1999). C to U editing of the anticodon of imported mitochondrial tRNA^Trp^ allows decoding of the UGA stop codon in *Leishmania tarentolae*. EMBO J..

[193] Aldinger C.A., Leisinger A.K., Gaston K.W., Limbach P.A., Igloi G.L. (2012). The absence of A-to-I editing in the anticodon of plant cytoplasmic tRNA^Arg(ACG)^ demands a relaxation of the wobble decoding rules. RNA Biol..

[194] Crain P.F., Alfonzo J.D., Rozenski J., Kapushoc S.T., McCloskey J.A., Simpson L. (2002). Modification of the universally unmodified uridine-33 in a mitochondria-imported edited tRNA and the role of the anticodon arm structure on editing efficiency. RNA.

[195] Bruske E.I., Sendfeld F., Schneider A. (2009). Thiolated tRNAs of *Trypanosoma brucei* are imported into mitochondria and dethiolated after import. J. Biol. Chem..

[196] Brindefalk B., Viklund J., Larsson D., Thollesson M., Andersson S.G. (2007). Origin and evolution of the mitochondrial aminoacyl-tRNA synthetases. Mol. Biol. Evol..

[197] Bonnefond L., Fender A., Rudinger-Thirion J., Giegé R., Florentz C., Sissler M. (2005). Towards the full set of human mitochondrial aminoacyl-tRNA synthetases: Characterization of AspRS and TyrRS. Biochemistry.

[198] Haen K.M., Pett W., Lavrov D.V. (2010). Parallel loss of nuclear-encoded mitochondrial aminoacyl-tRNA synthetases and mtDNA-encoded tRNAs in *Cnidaria*. Mol. Biol. Evol..

[199] Sanni A., Walter P., Boulanger Y., Ebel J.-P., Fasiolo F. (1991). Evolution of aminoacyl-tRNA synthetase quaternary structure and activity: *Saccharomyces cerevisiae* mitochondrial phenylalanyl-tRNA synthetase. Proc. Natl. Acad. Sci. USA.

[200] Klipcan L., Finarov I., Moor N., Safro M.G. (2010). Structural aspects of phenylalanylation and quality control in three major forms of phenylalanyl-tRNA synthetase. J. Amino Acids.

[201] Brandao M.M., Silva-Filho M.C. (2011). Evolutionary history of *Arabidopsis thaliana* aminoacyl-tRNA synthetase dual-targeted proteins. Mol. Biol. Evol..

[202] Duchêne A.-M., Giritch A., Hoffmann B., Cognat V., Lancelin D., Peeters N.M., Zaepfel M., Maréchal-Drouard L., Small I.D. (2005). Dual targeting is the rule for organellar aminoacyl-tRNA synthetases in *Arabidopsis thaliana*. Proc. Natl. Acad. Sci. USA.

[203] Bullard J.M., Cai Y.C., Spremulli L.L. (2000). Expression and characterization of the human mitochondrial leucyl-tRNA synthetase. Biochim. Biophys. Acta.

[204] Ibba M., Söll D. (2000). Aminoacyl-tRNA synthesis. Annu. Rev. Biochem..

[205] Ito T., Yokoyama S. (2010). Two enzymes bound to one tRNA assume alternative conformations for consecutive reactions. Nature.

[206] Pujol C., Bailly M., Kern D., Maréchal-Drouard L., Becker H., Duchêne A.-M. (2008). Dual-targeted tRNA-dependent amidotransferase ensures both mitochondrial and chloroplastic gln-tRNA^Gln^ synthesis in plants. Proc. Natl. Acad. Sci. USA.

[207] Fréchin M., Senger B., Braye M., Kern D., Martin R.P., Becker H.D. (2009). Yeast mitochondrial gln-tRNA^Gln^ is generated by a GATfab-mediated transamidation pathway involving ARC1p-controlled subcellular sorting of cytosolic GluRS. Genes Dev..

[208] Araiso Y., Huot J.L., Sekiguchi T., Frechin M., Fischer F., Enkler L., Senger B., Ishitani R., Becker H.D., Nureki O. (2014). Crystal structure of *Saccharomyces cerevisiae* mitochondrial GATfab reveals a novel subunit assembly in tRNA-dependent amidotransferases. Nucleic Acids Res..

[209] Nagao A., Suzuki T., Katoh T., Sakaguchi Y. (2009). Biogenesis of glutaminyl-mt tRNA^Gln^ in human mitochondria. Proc. Natl. Acad. Sci. USA.

[210] Echevarria L., Clemente P., Hernandez-Sierra R., Gallardo M.E., Fernandez-Moreno M.A., Garesse R. (2014). Glutamyl-tRNA^Gln^ amidotransferase is essential for mammalian mitochondrial translation *in vivo*. Biochem. J..

[211] Mailu B.M., Ramasamay G., Mudeppa D.G., Li L., Lindner S.E., Peterson M.J., DeRocher A.E., Kappe S.H., Rathod P.K., Gardner M.J. (2013). A nondiscriminating glutamyl-tRNA synthetase in the plasmodium apicoplast: The first enzyme in an indirect aminoacylation pathway. J. Biol. Chem..

[212] Kumazawa Y., Himeno H., Miura K.-I., Watanabe K. (1991). Unilateral aminoacylation specificity between bovine mitochondria and eubacteria. J. Biochem. (Tokyo).

[213] Fender A., Sauter C., Messmer M., Pütz J., Giegé R., Florentz C., Sissler M. (2006). Loss of a primordial identity element for a mammalian mitochondrial aminoacylation system. J. Biol. Chem..

[214] Charriere F., O’Donoghue P., Helgadottir S., Maréchal-Drouard L., Cristodero M., Horn E.K., Söll D., Schneider A. (2009). Dual targeting of a tRNA^Asp^ requires two different aspartyl-tRNA synthetases in *Trypanosoma brucei*. J. Biol. Chem..

[215] Fender A., Gaudry A., Jühling F., Sissler M., Florentz C. (2012). Adaptation of aminoacylation identity rules to mammalian mitochondria. Biochimie.

[216] Giegé R., Eriani G. (2014). Transfer RNA recognition and aminoacylation by synthetases. Encyclopedia of Life Sciences (ELS).

[217] Janke A., Pääbo S. (1993). Editing of a tRNA anticodon in marsupial mitochondria changes its codon recognition. Nucleic Acids Res..

[218] Giegé R., Sissler M., Florentz C. (1998). Universal rules and idiosyncratic features in tRNA identity. Nucleic Acids Res..

[219] Sohm B., Frugier M., Brulé H., Olszak K., Przykorska A., Florentz C. (2003). Towards understanding human mitochondrial leucine aminoacylation identity. J. Mol. Biol..

[220] Sohm B., Sissler M., Park H., King M.P., Florentz C. (2004). Recognition of human mitochondrial tRNA^Leu(UUR)^ by its cognate leucyl-tRNA synthetase. J. Mol. Biol..

[221] Goto Y., Nonaka I., Horai S. (1990). A mutation in the tRNA^Leu(UUR)^ gene associated with the MELAS subgroup of mitochondrial encephalomyopathies. Nature.

[222] van den Ouweland J.M.W., Lemkes H.H.P.J., Ruitenbeek W., Sandkuijl L.A., de Vijlder M.F. (1992). Mutation in mitochondrial tRNA^Leu(UUR)^ gene in a large pedigree with maternally transmitted type II diabetes mellitus and deafness. Nat. Genet..

[223] Florentz C., Sissler M., Lapointe J., Brakier-Gingras L. (2003). Mitochondrial tRNA aminoacylation and human diseases. Translation Mechanisms.

[224] Bonnefond L., Frugier M., Giegé R., Rudinger-Thirion J. (2005). Human mitochondrial TyrRS disobeys the tyrosine idenity rules. RNA.

[225] Bonnefond L., Giegé R., Rudinger-Thirion J. (2005). Evolution of the tRNA^Tyr^/TyrRS aminoacylation systems. Biochimie.

[226] Bonnefond L., Frugier M., Touzé E., Lorber B., Florentz C., Giegé R., Sauter C., Rudinger-Thirion J. (2007). Crystal structure of human mitochondrial tyrosyl-tRNA synthetase reveals common and idiosyncratic features. Structure.

[227] Jin X.L., Tao Z.J., Jia J., He X.X., Jin Y.X. (2006). Species-specific aminoacylation of *Oryza sativa* mitochondrial tRNA^Trp^. Chinese Sci. Bull..

[228] Shimada N., Suzuki T., Watanabe K. (2001). Dual mode of recognition of two isoacceptor tRNAs by mammalian mitochondrial seryl-tRNA synthetase. J. Biol. Chem..

[229] Sampson J.R., Saks M.E. (1993). Contributions of discrete tRNA^Ser^ domains to aminoacylation by *E. coli* seryl-tRNA synthetase: A kinetic analysis using model rna substrates. Nucleic Acids Res..

[230] Waeschenbach A., Porter J.S., Hughes R.N. (2012). Molecular variability in the *Celleporella hyalina* (*Bryozoa; Cheilostomata*) species complex: Evidence for cryptic speciation from complete mitochondrial genomes. Mol. Biol. Rep..

[231] Lovato M.A., Chihade J.W., Schimmel P. (2001). Translocation within the acceptor helix of a major tRNA identity determinant. EMBO J..

[232] Chihade J.W., Hayashibara K., Shiba K., Schimmel P. (1998). Strong selective pressure to use G:U to mark an RNA acceptor stem for alanine. Biochemistry.

[233] Aphasizhev R., Senger B., Rengers J.U., Sprinzl M., Walter P., Nussbaum G., Fasiolo F. (1996). Conservation in evolution for a small monomeric phenylalanyl-tRNA synthetase of the tRNA^Phe^ recognition nucleotides and initial aminoacylation site. Biochemistry.

[234] Tinkle-Peterson E., Uhlenbeck O.C. (1992). Determination of recognition nucleotides for *Escherichia coli* phenylalanyl-tRNA synthetase. Biochemistry.

[235] Giegé R., Lapointe J., Grosjean H. (2009). Transfer RNA aminoacylation and modified nucleosides. DNA and RNA Modification Enzymes: Structure, Mechanism, Function and Evolution.

[236] Renaud M., Ehrlich R., Bonnet J., Remy P. (1979). Lack of correlation between affinity of the tRNA for the aminoacyl-tRNA synthetase and aminoacylation capacity as studied with modified tRNA^Phe^. Eur. J. Biochem..

[237] Khvorova A.M., Motorin Y.A., Wolfson A.D., Gladilin K.L. (1992). Anticodon-dependent aminoacylation of RNA minisubstrate by lysyl-tRNA synthetase. FEBS Lett..

