# Correction: Salinas-Giegé et al. tRNA Biology in Mitochondria. *Int. J. Mol. Sci.* 2015, *16*, 4518–4559

**DOI:** 10.3390/ijms27010367

**Published:** 2025-12-29

**Authors:** Thalia Salinas-Giegé, Richard Giegé, Philippe Giegé

**Affiliations:** 1 Institut de Biologie Moléculaire des Plantes, Centre National de la Recherche Scientifique, Université de Strasbourg, 12 rue du Général Zimmer, F-67084 Strasbourg Cedex, France; thalia.salinas@ibmp-cnrs.unistra.fr; 2 Institut de Biologie Moléculaire et Cellulaire, Centre National de la Recherche Scientifique, Université de Strasbourg, 15 rue René Descartes, F-67084 Strasbourg Cedex, France; giegerichard@orange.fr

There was an error in the original publication [1]. The sentence “Similarly, in the moss *P. patens*, cytosolic tRNA^Arg(ACG)^ is imported in mitochondria where it undergoes an A to I editing at the wobble position of the anticodon [193]” on Paragraph 2 of Section 4.3.2 is incorrect.

A correction has been made to Section 4 ‘Biogenesis of Functional Mitochondrial tRNAs’, Section 4.3 ‘Functions and Mechanisms for tRNA Modifications and Editing’, Section 4.3.2 ‘Mitochondrial tRNA Editing’, Paragraph 2:

Beyond the mitochondrial encoded tRNAs, editing processes were also reported for cytosolic tRNAs imported into mitochondria. For instance, it was shown in *L. tarentolae* that a specific C to U editing in the anticodon of the imported nonedited tRNA^Trp^ allows the decoding of mitochondrial UGA codons as tryptophans [192]. In contrast, in the moss *P. patens* a tRNA^Arg(ACG)^ isoacceptor encoded in mitochondria, thus not imported from the cytosol, is deaminated at position A34 to obtain an ICG anticodon [193]. Interestingly for protozoan tRNA^Trp^, import and editing have drastic structural consequences, since this tRNA is partially thiolated at universally conserved position 33 to s^2^U33 and O′-methylated at the ribose moiety of Ψ32 as well as on edited U34 and nonedited C34 [194]. The presence of s^2^U33 and the concentration of O′-methylated pyrimidines in the anticodon domain, likely is the prerequisite for proper decoding of the Trp codon by conferring conformation rigidity and chemical stability to the anticodon loop. In contrast, dethiolation of cmm^5^s^2^U within mitochondria at wobble position 34 of imported trypanosomatid tRNAs, as explicitly shown for *T. brucei* tRNA^Glu^, is an alternate editing mechanisms required to modulate decoding [195].

The authors state that the scientific conclusions are unaffected. This correction was approved by the Academic Editor. The original publication has also been updated.
