# Supplementary material for: tRNA Biology in Mitochondria
Source: Int J Mol Sci. 2015 Feb 27;16(3):4518–59. doi: 10.3390/ijms16034518 (PMC4394434; doi:10.3390/ijms16034518)
Supplement: Supplementary file 1 [file ijms-16-04518-s001.pdf]

Supplementary Information

Table S1. Distribution of post-transcriptional modifications in mt-tRNAs from 10 phyla across phylogeny.

Nts	I<br>Pro.	II<br>Fun.	III<br>Nem.	IV<br>Mol.	V<br>Ech.	VI<br>Ins.	VII<br>Uro.	VIII<br>Bir.	IX<br>Mam.	X<br>Pla.	Σ
6	–	–	–	–	–	–	m^{2}G	–	m^{2}G	–	m^{2}G
8	–	–	m^{1}A	–	–	–	–	–	–	–	m^{1}A
9	–	–	m^{1}A	m^{1}A	m^{1}A<br>m^{1}G	m^{1}A<br>m^{1}G	m^{1}A	–	m^{1}A<br>m^{1}G	–	m^{1}A, m^{1}G
10	–	m^{2}G	–	–	m^{2}G	m^{2}G	m^{2}G	–	m^{2}G	–	m^{2}G
13	–	–	–	–	D	–	–	–	–	Ψ	Ψ
15	–	D	–	–	–	–	–	–	–	–	D
16	D	–	–	D	m^{1}A	–	–	–	–	D	D, m^{1}A
17	D	–	–	–	–	–	–	–	–	D	D
18	–	–	–	–	–	–	–	–	–	Gm	Gm
20	D	D	–	–	D	–	–	–	D	–	D
20a	–	D	–	–	–	–	–	–	–	D	D
20b	–	–	–	–	–	–	–	–	–	acp^{3}U	acp^{3}U
25	–	–	–	Ψ	–	–	Ψ	–	–	–	Ψ
26	m^{2}G<br>m$^2_2$G	m$^2_2$G	m^{2}G<br>m$^2_2$G, Ψ	–	–	Ψ	–	–	m^{2}G<br>m$^2_2$G	–	m^{2}G<br>m$^2_2$G, Ψ
27	Ψ	–	Ψ	Ψ	Ψ	Ψ	Ψ	Ψ	Ψ	Ψ	Ψ
28	–	–	Ψ	–	Ψ	Ψ	–	–	Ψ	–	Ψ
29	–	Ψ	Ψ, t^{6}A	–	–		–	–	Ψ	–	Ψ, t^{6}A
31	Ψ	Ψ	Ψ	–	–	Ψ	–	–	Ψ	–	Ψ
32	Ψm Cm	Ψ	Ψ	Ψ	Ψ, m^{3}C,<br>m^{5}C	Ψ	Ψ		Ψ, m^{3}C	–	Ψ, m^{3}C, Cm
33	s^{2}U	–	–	–	–	–	–	–	–	–	s^{2}U
34	Ψ, t^{6}A<br>cmnm^{5}U<br>cmnm^{5}s^{2}U<br>Um, Cm,	cmnm^{5}U	Ψ, f^{5}C<br>cmnm^{5}U<br>cmnm^{5}s^{2}U	m^{7}G	m^{7}G, Q	f^{5}C, Q	τm^{5}U<br>τm^{5}s^{2}U<br>(s^{2}U)	f^{5}C	f^{5}C, Q<br>τm^{5}U<br>τm^{5}s^{2}U	I, k^{2}C	Ψ, t^{6}A, f^{5}C,<br>m^{7}G, Q,<br>cmnm^{5}U,<br>τm^{5}s^{2}U<br>cmnm^{5}s^{2}U,<br>τm^{5}U<br>Um, Cm, I, k^{2}C
35	–	–	–	–	Ψ	–	–		–	–	Ψ
37	i^{6}A<br>t^{6}A<br>m^{1}G	i^{6}A	ms^{2}i^{6}A<br>m^{1}G<br>t^{6}A	t^{6}A	i^{6}A<br>t^{6}A,<br>m^{6}t^{6}A<br>m^{1}G	i^{6}A, t^{6}A<br>ms^{2}i^{6}A	ms^{2}i^{6}A		i^{6}A, t^{6}A<br>ms^{2}i^{6}A<br>m^{1}G	m^{6}A<br>m^{1}G	i^{6}A, m^{6}A, t^{6}A,<br>m^{6}t^{6}A, ms^{2}i^{6}A,<br>m^{1}G

Table S1. *Cont.*

Nts	I Pro.	II Fun.	III Nem.	IV Mol.	V Ech.	VI Ins.	VII Uro.	VIII Bir.	IX Mam.	X Pla.	Σ
38	–	–	–	–	Ψ, ms^{2}i^{6}A	Ψ	Ψ		–	Ψ	Ψ, ms^{2}i^{6}A
39	Ψ, Ψm	Ψ	–	Ψ	Ψ	Ψ, Ψm, Cm	Ψ	–	Ψ	Ψ	Ψ, Ψm, Cm
40	–	Ψ	–	–	Ψ	–	Ψ	–	Ψ	–	Ψ
41	Ψ	Ψ	–	–	–	Ψ	–	–	–	–	Ψ
44	Um	Um	–	–	–	Ψ	–	–	–	Um	Um
45	–	–	–	–	–	D	–	–	–	–	D
46	–	–	–	m^{7}G	–	–	–	–	–	m^{7}G	m^{7}G
47	–	–	–	D	–	–	–	–	–	acp^{3}U	D, acp^{3}U
48	–	–	–	m^{5}C	–	–	–	–	m^{5}C	–	m^{5}C
49	m^{5}C	–	–	–	–	–	–	–	m^{5}C	m^{5}C	m^{5}C
50	–	–	–	–	–	–	–	–	Ψ	–	Ψ
54	T	T	–	T	Ψ	–	–	T	–	T	T, Ψ
55	Ψ	Ψ	–	Ψ	Ψ	–	–	Ψ	Ψ	Ψ	Ψ
57	–	–	–	–	m^{1}A	–	–	–	Ψ	–	Ψ
58	–	–	–	–	–	–	–	–	m^{1}A	m^{1}A	m^{1}A
64							Ψ				Ψ
65	–	–	–	–	–	Ψ	–	–	–	–	Ψ
66	–	–	–	–	–	Ψ	–	–	–	–	Ψ
67	–	–	–	m^{2}G	–	Ψ	–	–	Ψ	–	m^{2}G, Ψ
68	–	–	–	–	–	Ψ	–	–	–	–	Ψ
70	–		Ψ	–	–	–	–	–	–	–	Ψ
71	–		Ψ	–	–	–	–	–	–	–	Ψ
72	–	–	–	m^{5}C	–	–	–	–	m^{5}C	–	m^{5}C
Nb Seq.	**4**	**25**	**10**	**4**	**7**	**16**	**6**	**1**	**35**	**15**	**123**

Most data are retrieved from *tRNAdb* data base [125] and additional data (in blue) are taken from refs [50,108,193,194,1,2]. The number of RNA sequences of mt-tRNAs investigated in 10 eukaryotic phyla is indicated. Modified nucleosides (nts) are found in Acceptor stem (nts 6 and 66–72), D-arm with connecting residues (nts 8–26), Anticodon loop (nts 32–38, on yellow background), Anticodon stem (nts 27–31 and 39–41, on light yellow background), Variable region (nts 44–48) and T-arm (nts 49–65); I Pro., unicellular protozoans including alveoletes, amoebas and euglenes (F, K, L, W, Y; *Leishmania tarentolae*, *Tetrahymena pyriformis*, *T. thermophila*, *Trypanosoma brucei*); II Fun., fungi (All but five—*C*, *D*, *E*, *N*; *Neurospora crassa*, *Saccharomyces cerevisiae*); III Nem., nematodes (F, E, K, L, M, Q, R, S, W; *Ascaris suum*); IV Mol., molluscs (K, S; *Loligo bleckeri*); V Ech., echinoderms (D, H, K, N, S, Y; *Asterias amurensis*); VI Ins., insects (D, E, G, I, K, M, N, Q, R, S, V, W; *Aedes albopictus*, *Drosophila melanogaster*); VII Uro., urochordates (G, M, W; *Halocynthia roretzi*); VIII Bir., birds (M; *Gallus gallus*); IX Mam., mammals (All; *Bos taurus*, *Didelphis virginiana*, *Homo sapiens*, *Mesocricetus auratus*, *Rattus norvegicus*); X Pla., plants (F, I, L, M, P, R, W, Y; *Oenothera*, *Phaseolus vulgaris*, *Physcomitrella patens*, *Solanum tuberosum*, *Triticum aestivum*). Σ The represented coding specificities are indicated by the amino acid one-letter code (missing specificities are in italics) and are scattered in the indicated species. § Data on *T. brucei* mt-tRNAs (K, L, W) [50] concern imported tRNAs modified in mitochondria to Cm32.

References

1. Tomita, K.; Ueda, T.; Watanabe, K. 7-Methylguanosine at the anticodon wobble position of squid mitochondrial $tRNA^{Ser(GCU)}$: Molecular basis for assignment of AGA/AGG codons as serine in invertebrate mitochondria. *Biochim. Biophys. Acta* **1998**, *1399*, 78–82.
2. Tomita, K.; Ueda, T.; Ishiwa, S.; Crain, P.F.; McCloskey, J.A.; Watanabe, K. Codon reading patterns in *Drosophila melanogaster* mitochondria based on their tRNA sequences: A unique wobble rule in animal mitochondria. *Nucleic Acids Res.* **1999**, *27*, 4291–4297.
